# Deep Learning Model for Predicting Immunotherapy Response in Advanced Non−Small Cell Lung Cancer

**DOI:** 10.1001/jamaoncol.2024.5356

**Published:** 2024-12-26

**Authors:** Mehrdad Rakaee, Masoud Tafavvoghi, Biagio Ricciuti, Joao V. Alessi, Alessio Cortellini, Fabrizio Citarella, Lorenzo Nibid, Giuseppe Perrone, Elio Adib, Claudia A. M. Fulgenzi, Cassio Murilo Hidalgo Filho, Alessandro Di Federico, Falah Jabar, Sayed Hashemi, Ilias Houda, Elin Richardsen, Lill-Tove Rasmussen Busund, Tom Donnem, Idris Bahce, David J. Pinato, Åslaug Helland, Lynette M. Sholl, Mark M. Awad, David J. Kwiatkowski

**Affiliations:** 1 Department of Medicine, Brigham and Women’s Hospital, Harvard Medical School, Boston, Massachusetts; 2 Department of Cancer Genetics, Oslo University Hospital, Oslo, Norway; 3 Department of Clinical Pathology, University Hospital of North Norway, Tromsø, Norway; 4 Department of Medical Biology, UiT The Arctic University of Norway, Tromsø, Norway; 5 Department of Community Medicine, UiT The Arctic University of Norway, Tromsø, Norway; 6 Lowe Center for Thoracic Oncology, Dana-Farber Cancer Institute, Harvard Medical School, Boston, Massachusetts; 7 Department of Surgery and Cancer, Imperial College London, London, United Kingdom; 8 Medical Oncology Operative Research Unit, Fondazione Policlinico Campus Bio-Medico, Rome, Italy; 9 Research Unit of Medical Oncology, Department of Medicine and Surgery, Universitá Campus Bio-Medico, Rome, Italy; 10 Research Unit of Anatomical Pathology, Department of Medicine and Surgery, Università Campus Bio-Medico, Rome, Italy; 11 Anatomical Pathology Operative Research Unit, Fondazione Policlinico Università Campus Bio-Medico, Rome, Italy; 12 Department of Pulmonary Medicine, Cancer Center Amsterdam, VU Medical Center, Amsterdam University Medical Center, Amsterdam, the Netherlands; 13 Department of Clinical Medicine, UiT The Arctic University of Norway, Tromsø, Norway; 14 Department of Oncology, University Hospital of North Norway, Tromsø, Norway; 15 Department of Translational Medicine, University of Piemonte Orientale, Novara, Italy; 16 Division of Clinical Medicine, University of Oslo, Oslo, Norway; 17 Department of Pathology, Brigham and Women’s Hospital, Harvard Medical School, Boston, Massachusetts; 18 Department of Medical Oncology, Dana-Farber Cancer Institute, Boston, Massachusetts

## Abstract

**Importance:**

Only a small fraction of patients with advanced non−small cell lung cancer (NSCLC) respond to immune checkpoint inhibitor (ICI) treatment. For optimal personalized NSCLC care, it is imperative to identify patients who are most likely to benefit from immunotherapy.

**Objective:**

To develop a supervised deep learning−based ICI response prediction method; evaluate its performance alongside other known predictive biomarkers; and assess its association with clinical outcomes in patients with advanced NSCLC.

**Design, Setting, and Participants:**

This multicenter cohort study developed and independently validated a deep learning−based response stratification model for predicting ICI treatment outcome in patients with advanced NSCLC from whole slide hematoxylin and eosin–stained images. Images for model development and validation were obtained from 1 participating center in the US and 3 in the European Union (EU) from August 2014 to December 2022. Data analyses were performed from September 2022 to May 2024.

**Exposure:**

Monotherapy with ICIs.

**Main Outcomes and Measures:**

Model performance measured by clinical end points and objective response rate (ORR) differentiation power vs other predictive biomarkers, ie, programmed death-ligand 1 (PD-L1), tumor mutational burden (TMB), and tumor-infiltrating lymphocytes (TILs).

**Results:**

A total of 295 581 image tiles from 958 patients (mean [SD] age, 66.0 [10.6] years; 456 [48%] females and 502 [52%] males) treated with ICI for NSCLC were included in the analysis. The US-based development cohort consisted of 614 patients with median (IQR) follow-up time of 54.5 (38.2-68.1) months, and the EU-based validation cohort, 344 patients with 43.3 (27.4-53.9) months of follow-up. The ORR to ICI was 26% in the developmental cohort and 28% in the validation cohort. The deep learning model’s area under the receiver operating characteristic curve (AUC) for ORR was 0.75 (95% CI, 0.64-0.85) in the internal test set and 0.66 (95% CI, 0.60-0.72) in the validation cohort. In a multivariable analysis, the deep learning model’s score was an independent predictor of ICI response in the validation cohort for both progression-free (hazard ratio, 0.56; 95% CI, 0.42-0.76; *P* < .001) and overall survival (hazard ratio, 0.53; 95% CI, 0.39-0.73; *P* < .001). The tuned deep learning model achieved a higher AUC than TMB, TILs, and PD-L1 in the internal set; in the validation cohort, it was superior to TILs and comparable with PD-L1 (AUC, 0.67; 95% CI, 0.60-0.74), with a 10-percentage point improvement in specificity. In the validation cohort, combining the deep learning model with PD-L1 scores achieved an AUC of 0.70 (95% CI, 0.63-0.76), outperforming either marker alone, with a response rate of 51% compared to 41% for PD-L1 (≥50%) alone.

**Conclusions and Relevance:**

The findings of this cohort study demonstrate a strong and independent deep learning−based feature associated with ICI response in patients with NSCLC across various cohorts. Clinical use of this deep learning model could refine treatment precision and better identify patients who are likely to benefit from ICI for treatment of advanced NSCLC.

## Key Points

**Question:**

Can deep learning−based algorithms use histologic images to directly predict response to immune checkpoint inhibitors (ICI) in patients with advanced non−small cell lung cancer (NSCLC)?

**Findings:**

This cohort study developed and externally validated a response prediction computational pipeline including 958 patients with NSCLC treated with ICI monotherapy, and demonstrated that deep learning prediction scores were associated with response rate, progression-free survival, and overall survival, with performance comparable to programmed death-ligand 1 (PD-L1). Combined deep learning and PD-L1 scores improved patient stratification.

**Meaning:**

These findings indicate that an artificial intelligence pathology model could potentially serve as a new tool for guiding ICI treatment, refining patient selection, and improving clinical outcomes in the treatment of advanced NSCLC.

## Teaser

This multicenter cohort study develops and assesses the validity of a deep learning model for predicting immune checkpoint inhibitor response in patients with advanced non−small cell lung cancer.

## Introduction

Treatment with immune checkpoint inhibitors (ICIs) has shown clinical benefit for patients with advanced or metastatic non−small cell lung cancer (NSCLC) without *EGFR* or *ALK* alterations; however, just 25% to 30% will respond.^1,2^ The primary predictive biomarker of response to ICI monotherapy is programmed death-ligand 1 (PD-L1) protein expression,^3,4^ an imperfect measure given that some patients with low PD-L1 levels do benefit from ICI therapy, whereas not all those with high PD-L1 respond.^5,6^ In 2020, the US Food and Drug Administration approved tissue-derived tumor mutational burden (TMB) as a predictive biomarker for ICI in various solid tumors, including NSCLC.^7^ However, use of TMB faces challenges including cost, assay variability, defining optimal cutoff, and limited sensitivity and specificity.^8,9,10^ Consequently, there is continuing interest in identifying additional biomarkers for immunotherapy response in patients with advanced-stage cancer. ICI response can be reduced by some genomic variations (eg, *KEAP1*, *STK11*),^11^ and are associated with microsatellite instability,^12^ neoantigen load,^13^ tumor-inflamed phenotypes,^14^ tumor-infiltrating lymphocytes (TILs),^15^ and tertiary lymphoid structures (TLS).^16^

Recent developments in artificial intelligence have transformed computational pathology. Both machine learning and deep learning algorithms are used to analyze digital pathologic images, handling tasks such as tumor segmentation, grading, subtyping, and cell classification.^17^ We have previously developed several machine learning−based computational pathology classification systems, designed to identify immune phenotypes,^18^ TILs,^15^ and TLS^19^ using standard histologic digital images. The immune biomarkers derived from these machine learning processes have shown an association with the response to ICI monotherapy and overall survival in NSCLC and melanoma.^15,20^ In addition, they have been associated with the risk of recurrence in early-stage lung cancer.^18^

Deep learning models have been developed by several groups^21,22,23^ to interpret complex spatial patterns in histologic images and to predict factors such as survival and genomic alterations, at a level of sophistication beyond that of most human experts. The capability of deep learning to fully analyze image features, without prior constraint or bias, enables comprehensive assessment of many histopathologic patterns, potentially leading to more accurate predictions of clinical outcomes.

Extending these efforts, we sought to develop a deep learning−based response stratification model to directly predict ICI efficacy from digital images of pathology specimens in patients with advanced NSCLC. We aimed to externally validate the model in a large independent cohort and to compare its outputs with PD-L1, TMB, and TIL levels for predicting response to ICI treatment.

## Methods

The institutional review board at each of the 4 participating institutions granted approval for the retrospective collection of patient datasets in each cohort. A comprehensive description of the methods is available in the eMethods in Supplement 1. This multicenter study was conducted across continents with varying ethical requirements. All patients provided informed consent, except for the UK cohort, where consent was waived due to the retrospective nature of the data collection. The study followed the REMARK reporting guideline.

### Study Design and Dataset Selection

This multicenter cohort study was conducted at 1 center in the US and 3 centers in the European Union (EU) from February 2014 to December 2022. The medical records and histologic images of patients with NSCLC treated with ICIs alone (without chemotherapy), either as first-line or subsequent-line therapy, were used for model development and validation. We included 1135 eligible participants and excluded 177 for reasons detailed in the eMethods in Supplement 1.

#### Developmental Cohort

The developmental cohort included consecutive patients with histologically confirmed advanced or metastatic stage NSCLC who underwent targeted next-generation sequencing and treated with ICIs by the Dana-Farber Cancer Institute (DFCI; Boston, Massachusetts) from August 2014 to May 2022. This cohort was divided into 2 groups, 1 for training and development of the deep learning model, and the other for testing and evaluating its performance.

#### Validation Cohort

To test its generalizability, the deep learning model was validated among patients with advanced or metastatic stage NSCLC treated with ICIs at 3 centers in the EU. This cohort included patients treated by the Fondazione Policlinico Universitario Campus Bio-Medico at the University of Rome (FPUCBM; Italy) from May 2016 to December 2022; the Amsterdam University Medical Center (AUMC; the Netherlands) from January 2015 to September 2021; and the Imperial College of London (ICL; United Kingdom) from February 2014 to August 2021.

### Study Procedures

After preprocessing whole slide images from hematoxylin-eosin (H&E) stains of surgical or biopsy specimens, we designed and built a supervised deep learning model, that we termed the *Deep-IO*. It was designed specifically for predicting responses to ICI monotherapy directly from histologic images. The model was trained based on the objective response rate (ORR) of ICI, as defined by RECIST (Response Evaluation Criteria in Solid Tumors), version 1.1 (eFigures 1 and 2 in Supplement 1).

In the DFCI cohort, TMB was defined as the total count of nonsynonymous missense variants and small insertion-deletion variants for each megabase in the genome sequenced, using the OncoPanel-Next-Generation Sequencing test.^24^ The PD-L1 tumor proportion score (TPS) was calculated based on the proportion of tumor cells with any PD-L1 expression in samples with at least 100 viable tumor epithelial cells. For the categorization of whole slide H&E images into TILs, tumor cells, or stromal cells, we used a machine learning model utilizing the random forest algorithm.^15^ TILs were recognized as mononuclear immune cells including lymphocytes and plasma cells. An overview of the data availability on PD-L1, TMB, and TILs in both cohorts is shown in eFigure 3 in Supplement 1.

### Statistical Analysis

Deep-IO probability scores were categorized using median and tertile cutoffs from the validation cohort for survival analysis. Mann-Whitney U tests were used for comparing continuous variables between 2 groups; Spearman correlation for 2 continuous variables; and χ^2^ tests for associations between categorical variables. Kaplan-Meier and log-rank tests were used for survival analysis, and hazard ratios (HRs) were derived from univariate and multivariable Cox models. Receiver operating characteristic curve analysis provided area under the receiver operating characteristic curve (AUC), sensitivity, specificity, PPV, and NPV for Deep-IO, TMB, PD-L1, and TILs, and Deep-IO and PD-L1 continuous scores were combined through logistic regression. Additional details are available in the eMethods in Supplement 1. Statistical tests were 2-tailed, and *P* < .05 was considered statistically significant. Data analyses were performed from September 2022 to May 2024 using R, version 4.3.1 (R Foundation for Statistical Computing), and Python.

## Results

### Clinical Features of the Datasets

Deep-IO was trained and validated on 295 581 image tiles (512 × 512 pixels), after quality control (eFigure 4 and eMethods in Supplement 1), from 958 patients (mean [SD] age, 66.0 [10.6] years; 456 [48%] females and 502 [52%] males); 156 969 tiles from the 614 patients in the US cohort (DFCI) for model training and testing, and 138 612 tiles from the 344 patients in the EU cohort (137 patients from FPUCBM; 130 from AUMC; and 77 from ICL) were used for external and independent validation (eFigure 5 in Supplement 1). The development cohort was divided as follows: 85% (n = 521) for training and development of the deep learning model, and 15% (n = 93) for testing and evaluating its performance. The baseline patient and tumor characteristics of the developmental and validation cohorts were heterogeneous in terms of ICI agent; sex; histologic findings; tumor, sample type, and site; and *EGFR* and *KRAS* status; however, they were similar in risk factors that might influence ICI response—eg, treatment line, Eastern Cooperative Oncology Group (ECOG) performance status, age, and high (≥50%) PD-L1 expression (Table 1).

**Table 1.** Clinical Characteristics of the 2 Cohorts Used to Develop and Validate the Deep-IO Model

Characteristic	No. (%)		*P* value
	Developmental cohort (n = 614)	External validation cohort (n = 344)	
Institute, country			
DFCI, US	614 (100)	NA	NA
FPUCBM, Italy	NA	137 (40)	
AUMC, Netherlands	NA	130 (38)	
ICL, United Kingdom	NA	77 (22)	
Immune checkpoint inhibitors			
Pembrolizumab	351 (57)	183 (53)	< .001
Nivolumab	201 (32)	121 (35)	
Atezolizumab	34 (5)	38 (11)	
Durvalumab	1 (<1)	2 (1)	
Nivolumab + ipilimumab	19 (3)	NA	
Pembrolizumab + ipilimumab	6 (1)	NA	
Tremelimumab + durvalumab	2 (<1)	NA	
Line of therapy			
1	268 (44)	161 (47)	.30
≥2	346 (56)	183 (53)	
Age, median (range), y	67 (27-92)	68 (37-94)	.70
Sex			
Female	337 (55)	119 (35)	< .001
Male	277 (45)	225 (65)	
Histology			
LUAD	479 (78)	237 (69)	.01
LUSC	89 (14)	77 (22)	
Other	46 (7)	30 (9)	
Smoking			
Never	78 (13)	30 (9)	.09
Ever	536 (87)	310 (90)	
Unknown		4 (1)	
ECOG PS			
0-1	498 (81)	290 (84)	.10
≥2	112 (18)	48 (14)	
Unknown	4 (1)	6 (2)	
Specimen site			
Lung	310 (51)	211 (61)	< .001
Lymph node	81 (13)	34 (10)	
Pleura	54 (9)	16 (5)	
Brain	53 (8)	7 (2)	
Liver	35 (6)	28 (8)	
Soft tissue	35 (6)	14 (4)	
Other^a^	46 (7)	34 (10)	
Tumor type			
Primary	138 (23)	203 (59)	< .001
Metastatic	462 (75)	141 (41)	
Unknown	14 (2)		
Tissue type			
Surgery	176 (29)	70 (20)	.005
Biopsy	438 (71)	274 (80)	
*KRAS*/*EGFR* status			
* KRAS*	227 (37)	177 (51)	< .001
* EGFR*	51 (8)	5 (2)	
*KRAS/EGFR* neg	336 (55)	123 (36)	
Not tested	NA	39 (11)	
PD-L1 (TPS %)			
<1	77 (13)	75 (22)	< .001
1-49	154 (25)	61 (17)	
≥50	247 (40)	149 (43)	
Unknown	136 (22)	59 (17)	
TMB (mu/Mb)			
<10	316 (51)	6 (2)	NA
≥10	295 (48)	13 (4)	
Unknown	3 (<1)	325 (94)	

Abbreviations: AUMC, Amsterdam University Medical Center; DFCI, Dana Farber Cancer Institute; ECOG PS, Eastern Cooperative Oncology Group performance score; FPUCBM, Fondazione Policlinico Universitario Campus Bio-Medico, University of Rome; ICI, immune checkpoints inhibitor; ICL, Imperial College London; LUAD, lung adenocarcinoma; LUSC, lung squamous cell carcinoma; mu/Mb, mutations per megabase; NA, not applicable; TMB, tumor mutational burden; TPS, tumor proportion score.

^a^ Including breast, skin, kidney, stomach, small intestine, adrenal, and oral tissues.

The model was trained to predict the ORR, classifying responses as either responders (complete response [CR] and partial response [PR]) or nonresponders (progressive disease [PD] and stable disease [SD]). In the DFCI cohort, the ORR was 26% (159 of 614 patients). In the EU validation cohort, the ORR was 28% (96 of 344 patients; eFigure 6 in Supplement 1).

### Deep Learning Model Performance and Interpretability

The Deep-IO model assigned a prediction probability from 0 to 1 to each image tile, indicating the likelihood of ICI therapy response; scores less than 0.5 suggested nonresponders and scores of 0.5 or greater indicated responders. We computed the patient-level Deep-IO score by averaging the tile scores, applying the same classification criteria across both test and external validation cohorts for performance metric evalution.^25,26^

Given that predictions primarily occur at the image tile level, we explored whether the number of tiles per patient affected the patient-level Deep-IO score. We found no correlation between the number of tiles and the Deep-IO score in surgical or biopsy specimens from either cohort (eFigure 7 in Supplement 1).

The test set from DFCI included 93 patients. In predicting the ICI response, Deep-IO achieved an F1-score of 0.71; recall, 0.70; and precision, 0.73. In the independent validation cohort, the model demonstrated comparable performance, with an F1-score of 0.69; recall, 0.71; and precision, 0.69. Performance metrics within the subcohorts of the validation set were consistent with the overall validation cohort performance, except in the ICL cohort (n = 77), which showed a slightly lower performance (eFigure 8 in Supplement 1).

For the DFCI test set, the Deep-IO model correctly predicted nonresponder status for 51 of 69 patients (55%) and responder status for 14 of 24 patients (15%), achieving an overall accuracy of 70%. In the external validation cohort, the model accurately identified 217 of 248 nonresponders (63%) and 28 of 96 responders (8%), with a total accuracy of 71% (eFigure 9 in Supplement 1).

For model explainability, we used Gradient-weighted Class Activation Mapping (GradCam) to highlight important subregions at the whole slide level (eMethods in Supplement 1). Pathologist assessed model focus areas (tumor, stroma, inflammatory reaction) semiquantitatively on whole slide imaging from 25 randomly selected patients per response category. The model primarily focused on tumor epithelial compartments, scoring 2.3 for responders and 2.1 for nonresponders, followed by inflammatory areas (responders = 1.8; nonresponders = 1.4) and stroma (responders = 1.2; nonresponders = 1.0). eFigure 10 in Supplement 1 shows these spatial predictions through GradCam.

### Deep Learning Model and ICI Clinical End Points

**Figure 1.**
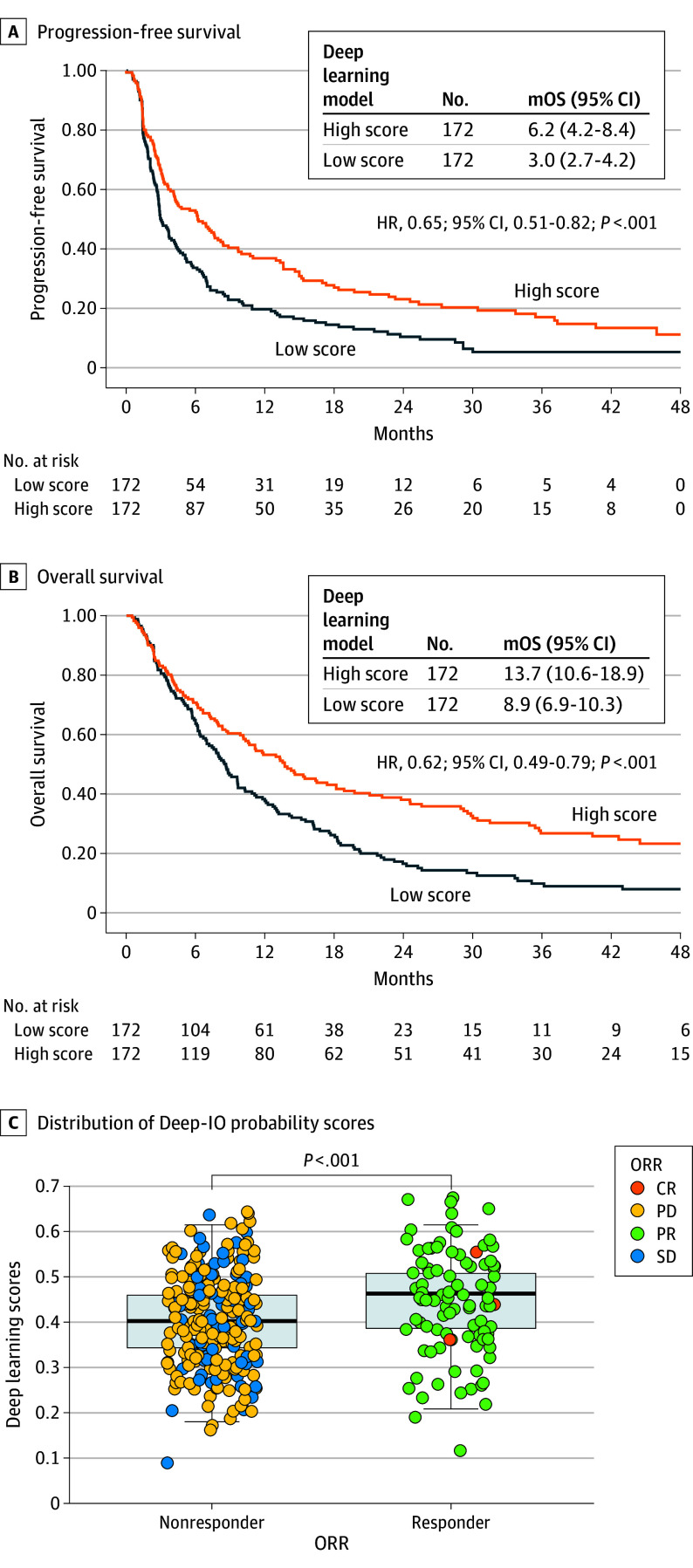
Deep Learning Model and Immune Checkpoint Inhibitors (ICIs) by Clinical Outcome A and B, Progression-free survival and overall survival in response to ICI, stratified by the Deep-IO model scores in the validation cohort. C, Distribution of Deep-IO probability scores across ORR subgroups in the validation cohort using the Mann-Whitney U-test. CR indicates complete response; HR, hazard ratio; mOS, median OS in months; mPFS, median PFS in months; ORR, objective response rate; PD, progressive disease; PR, partial response; and SD, stable disease.

The study’s median (IQR) follow-up was 54.5 months (38.2-68.1) in the developmental cohort and 43.3 months (27.4-53.9) in the validation cohort. Median (IQR) progression-free survival (PFS) was 3.7 months (1.7-10.1) and 4.2 months (1.9-14.5), respectively. Because most (85%) cases in the developmental cohort were used for training, the association of the Deep-IO score with ICI outcome data was primarily evaluated in the validation cohort. Patients with higher Deep-IO scores (>median) had significantly longer PFS and overall survival (OS) (Figure 1A and B). As a continuous variable, the Deep-IO score was higher in the CR and PR (responders) group compared to the SD and PD group (nonresponders; Figure 1C).

Considering statistical power, PFS and ORR results were consistent in the validation subcohorts (FPUCBM and ICL), except the AUMC dataset showed a nonsignificant trend toward higher median PFS (7.2 vs 3.6 months; *P* = .45) associated with the Deep-IO score (eFigure 11 in Supplement 1).

Patients receiving first-line ICI treatment had higher response rates (32% and 37.9%) than those on subsequent-line treatment (21.1% and 19.1%) in both cohorts (eFigure 12 in Supplement 1). In the validation cohort, the Deep-IO score was significantly associated with survival in both treatment lines (eFigure 13 in Supplement 1). Subgroup analysis showed that the Deep-IO association was consistent in the anti-PD1 group (pembrolizumab/nivolumab; n = 304) for PFS and OS; however, in the anti-PD-L1 group (atezolizumab/durvalumab; n = 40), it was limited to OS (eFigure 14 in Supplement 1).

After stratification based on the main histologic subtypes in the validation cohort, the Deep-IO score showed an association with ICI for PFS (95% CI, 0.40-0.72; *P* < .001) and OS (95% CI, 0.38-0.69; *P* < .001) in the lung adenocarcinoma (n = 237) subgroup, while no significant association with PFS and OS was observed in patients with lung squamous cell carcinoma (n = 77; eFigure 15 in Supplement 1).

**Figure 2.**
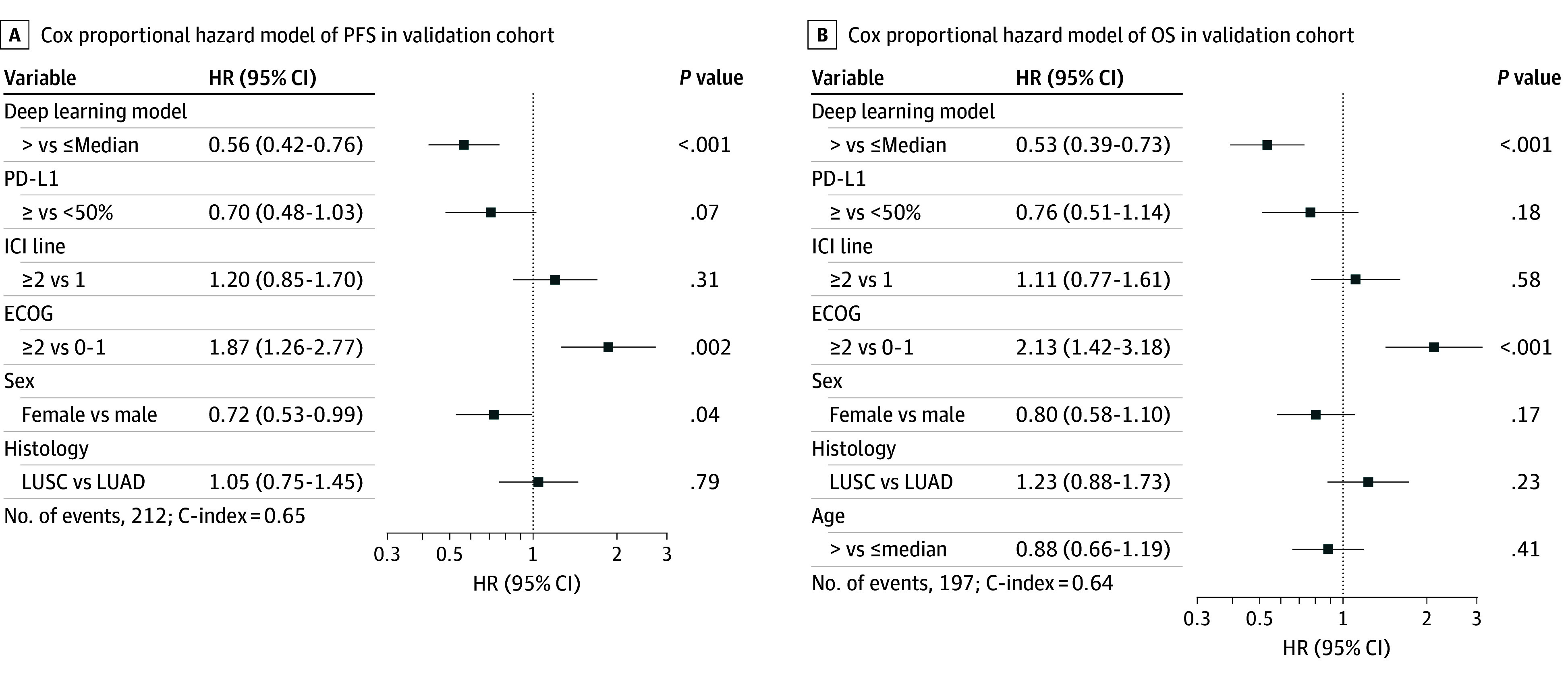
Multivariable Analysis in the Validation Cohort Cox proportional hazard models of significant independent predictive factors associated with progression-free survival and overall survival. Variables with *P* < .25 from univariate analyses were included in the Cox regression analysis. Number of events and C-index are indicated for each model. C-index indicates concordance index; ECOG, Eastern Cooperative Oncology Group performance status; ICI, immune checkpoint inhibitors; LUAD, lung adenocarcinoma; and LUSC, lung squamous cell carcinoma.

Multivariable analysis included all covariates that were significant in the univariate analysis: PD-L1, ICI line, ECOG status, sex, histologic findings, and age (only for OS) (eFigures 16 and 17 in Supplement 1). Deep-IO was an independent predictive factor for both PFS (HR, 0.56; concordance index, 0.65) and OS (HR, 0.53; concordance index, 0.64; Figure 2).

### Deep Learning Model Across PD-L1 TPS Subgroups

The validation cohort was divided based on PD-L1 (TPS) to assess how the model’s effectiveness compared to TILs (per mm^2^) in these groups. In subsets with high PD-L1 (≥50%; n = 149) and moderate PD-L1 (1%-49%; n = 61), Deep-IO was superior to TILs in predicting ORR, with AUC scores of 0.63 (95% CI, 0.54-0.72) for high PD-L1 and 0.74 (95% CI, 0.57-0.87) for moderate PD-L1 (eFigure 18 in Supplement 1). However, this was not observed in the PD-L1−negative (<1%) subgroup. Similar findings were made considering PFS, with a significant association between Deep-IO score and PFS. In PD-L1 negative (n = 75), TILs/mm^2^ (AUC = 0.77) were more effective than Deep-IO (AUC = 0.53) in distinguishing ICI responders (eFigure 18 in Supplement 1).

### Deep Learning Model vs Other Known ICI Response Biomarkers

Across both the development and validation cohorts, the median (IQR) values for known markers were as follows: TMB (mutations per megabase), 9.88 (6.84-13.68); TILs, 359 (169-744); and PD-L1, 50 (2-80). We observed a low to moderate correlation between Deep-IO score and both TILs (*r* = 0.16; *P* < .001) and PD-L1 (*r* = 0.37; *P* < .001). However, no correlation was found between TMB/Mb and Deep-IO (eFigure 19 in Supplement 1).

**Figure 3.**
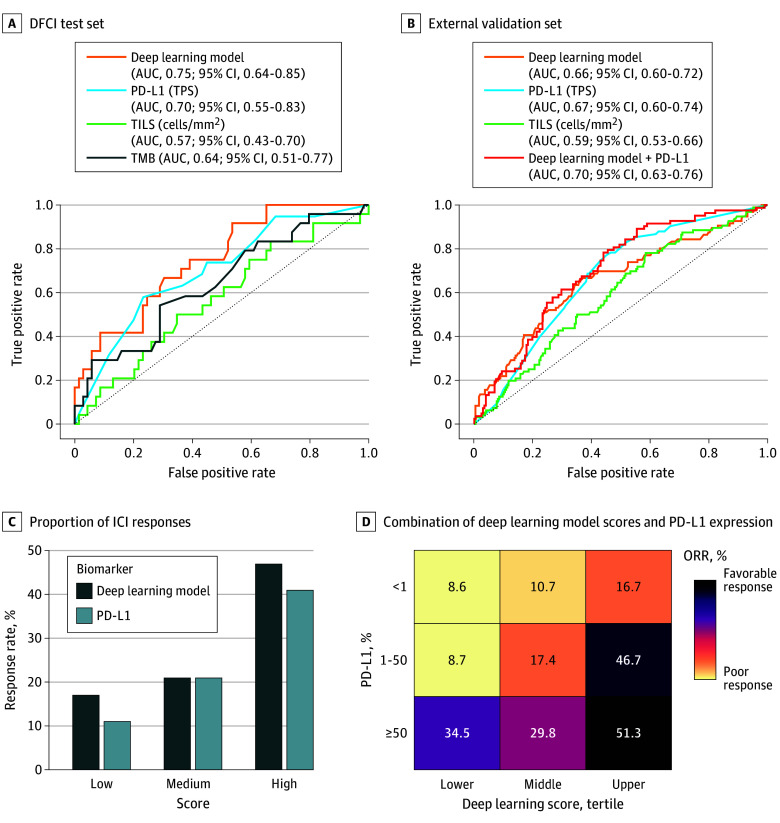
Performance of Immune Checkpoint Inhibitors (ICIs) Biomarkers vs the Deep-IO Model Analysis of the performance power of individual biomarkers (Deep-IO, PD-L1, TMB, TILs) and the combined Deep-IO and PD-L1 in differentiating ICI objective response rate binary groups in the A, test and B, validation cohorts. C, Proportion of ICI responses in PD-L1 and Deep-IO subgroups within the validation cohort, with Deep-IO subgroups classified into tertiles as low (lower tertile), medium (middle tertile), and high (upper tertile). PD-L1 subgroups are categorized as low (<1%), medium (1%-49%), and high (≥50%). D, Combination of Deep-IO scores and PD-L1 expression subgroups in relation to the ICI response rate within the validation cohort. The color intensity of the squares represents the response rate, with darker colors indicating a higher response rate and lighter colors indicating a lower response rate (shown as percentages). AUC indicates area under the receiver operating characteristic curve; HR, hazard ratio; ORR, objective response rate; PD-L1, programmed death-ligand 1; TILs, tumor-infiltrating lymphocytes; TMB, tumor mutational burden; and TPS, tumor proportion score.

To evaluate biomarker contributions in predicting ICI treatment ORR, we performed a receiver operating characteristic analysis using each biomarker as a continuous variable. In the test set (n = 93), Deep-IO had the highest AUC (0.75; 95% CI, 0.62-0.85) and sensitivity (0.91; 95% CI, 0.73-0.99), outperforming PD-L1, TILs, and TMB (Figure 3A; Table 2). In the validation cohort (n = 344), the AUC for Deep-IO (0.66; 95% CI, 0.60-0.72) was similar to that of PD-L1 (0.67; 95% CI, 0.60-0.74), but with 10% higher specificity for identifying nonresponders. Combining Deep-IO and PD-L1 scores via weighted regression improved response classification (AUC, 0.70; 95% CI, 0.63-0.76) and yielded the highest positive predictive value (0.42) and negative predictive value (0.86) compared to individual biomarkers (Figure 3B; Table 2).

**Table 2.** Comparative Performance of Various Biomarkers and Deep-IO for Identifying ICI Responders vs Nonresponders in the Test Set (n = 93) and External Validation Cohort (n = 344)

Biomarker	Value (95% CI)			
	Sensitivity	Specificity	PPV	NPV
**Test set**				
Deep-IO	0.91 (0.73-0.99)	0.47 (0.34-0.59)	0.37 (0.26-0.84)	0.94 (0.78-0.96)
PD-L1 (TPS %)	0.57 (0.33-0.79)	0.77 (0.64-0.86)	0.44 (0.29-0.70)	0.85 (0.68-0.91)
TILs (cells/mm^2^)	0.83 (0.62-0.95)	0.33 (0.22-0.46)	0.30 (0.20-0.64)	0.85 (0.66-0.90)
TMB (mu/Mb)	0.54 (0.32-0.74)	0.71(0.58-0.81)	0.40 (0.27-0.61)	0.82 (0.64-0.88)
**Validation cohort**				
Deep-IO	0.66 (0.55-0.75)	0.64 (0.58-0.70)	0.41 (0.35-0.53)	0.82 (0.76-0.86)
PD-L1 (TPS %)	0.77 (0.66-0.85)	0.54 (0.47-0.62)	0.41 (0.34-0.55)	0.85 (0.77-0.88)
TILs (cells/mm^2^)	0.78 (0.68-0.86)	0.41(0.35-0.48)	0.34 (0.28-0.46)	0.83 (0.74-0.86)
Deep-IO+PD-L1	0.78 (0.68-0.86)	0.56 (0.49-0.63)	0.42 (0.35-0.57)	0.86 (0.79-0.90)

Abbreviations: mu/Mb, mutations per megabase; NPV, negative predictive value; PPV, positive predictive value; TILs, tumor-infiltrating lymphocytes; TMB, tumor mutational burden; TPS, tumor proportion score.

Deep-IO scores were divided into tertiles (lower ≤0.37; middle = 0.37-0.46; upper ≥0.46) for equitable comparison with corresponding PD-L1 subgroups. The response rate in the upper Deep-IO tertile was 47%, higher than the 41% in the high PD-L1 (≥50%) group (Figure 3C). Combining Deep-IO with PD-L1 improved ORR stratification, with 51.3% responders in the high/high group and only 8.6% in the low/low group (Figure 3D). Additionally, Deep-IO maintained a significant association with a stepwise increase in median PFS from the lower to the upper tertile, mirroring the pattern observed with PD-L1 (eFigure 20 in Supplement 1).

To further assess the efficacy of the Deep-IO model in predicting response rates, we divided the validation cohort into subcohorts and compared them against established predictive biomarkers. The ORR for each subset was as follows: FPUCBM, 30.7%; AUMC, 20.8%; and ICL, 35.1% (eFigure 21 in Supplement 1). TMB data was limited, available for only a few cases (n = 19) in the FPUCBM subset, with an AUC of 0.56 (95% CI, 0.27-0.84). Comparing the AUC of Deep-IO with that of PD-L1 for ORR classification in each center yielded the following results: FPUCBM, 0.68 (95% CI, 0.58-0.79) vs 0.70 (95% CI, 0.59-0.79); AUMC, 0.57 (95% CI, 0.44-0.70) vs 0.64 (95% CI, 0.50-0.78); and ICL, 0.65 (95% CI, 0.52-0.76) vs 0.63 (95% CI, 0.50-0.76). TILs generally showed less performance power, except in the AUMC subset, where it achieved an AUC of 0.68 (95% CI, 0.57-0.78; eFigure 21 in Supplement 1).

## Discussion

This is the first proof-of-concept study to devise an artificial intelligence−driven model for predicting ICI response in advanced and metastatic stages of NSCLC using digital H&E pathology images. Across datasets from 1 US and 3 EU-based centers and various slide scanners, Deep-IO analysis demonstrated robust performance in predicting clinical outcomes of ICI therapy for patients treated in both first-line and subsequent lines. Deep-IO surpassed the predictive accuracy of established biomarkers such as machine learning−based TIL density and tissue TMB, and also showed significantly better performance in terms of HRs in multivariable analysis compared to PD-L1. The combination of Deep-IO and PD-L1 proved to be more effective in distinguishing between ICI responders and nonresponders than either assessment alone.

### Deep Learning Model’s Performance and Explainability

Deep-IO was developed using a supervised deep learning approach, leveraging a substantial dataset from the US. To ensure model generalizability, it underwent external validation with images and patient data from 3 centers in 3 different EU countries. The overall datasets incorporate histologic images acquired by various scanners and include patients from diverse backgrounds. Unlike other studies that have used machine or deep learning models for feature extraction and classification of specific biomarkers such as TILs and PD-L1 expression,^27,28^ we focused on directly predicting ICI response from histologic images using ORR as the ground truth. The ICI response rates were consistent across both the developmental and external validation cohorts, showing slight variation (26% vs 28%, respectively). Although the model training was based on binary labels of ICI response rates and demonstrated an association with ORR, we noted that increasing Deep-IO scores (categorized into tertiles) were correlated with better median PFS outcomes for ICI treatments (eFigure 20 in Supplement 1). This stepwise increase in scores highlights its clinical significance, effectively minimizing the risk of type I and II errors and enhancing the model’s reliability for clinical application.

In advanced NSCLC treated with ICIs, most patients have adenocarcinoma. Our subgroup analysis found that Deep-IO was associated with response in adenocarcinoma but not in squamous cell carcinoma (n = 77), likely due to the smaller sample size. Because Deep-IO is primarily informed by adenocarcinoma cases, its findings may not apply to squamous cell carcinoma, warranting further studies focused on this histology. Moreover, although Deep-IO demonstrated superior performance over TMB, achieving an AUC of 0.75 compared to 0.64 for predicting ICI outcomes in the developmental set, the absence of TMB data in the validation cohort calls for further confirmation through external datasets.

Deep learning models are frequently labeled “black-box” because their algorithms, which are trained instead of being directly coded, operate in a manner that is not transparent, making it challenging for humans to understand the rationale behind the outcomes. Using visualization tools has become a standard practice in the field to enhance model explainability.^29,30^ Our application of GradCam was intended to highlight which parts of an image are critical for the classification process in deep learning.^31,32,33^ In this context, and through a semiquantitative assessment of model focus areas for a subset of patients, we observed that the model predominantly directed its attention to tumor epithelial and inflammatory reaction subregions. This observation was consistent with our correlation analysis, which revealed a low to moderate association between the Deep-IO score and factors such as PD-L1 expression and immune cell infiltration. These results suggest that the model may be identifying 1 or more immunological features to inform its predictions.

### Deep Learning Model and PD-L1

In advanced-stage NSCLC, 2 standard treatments are commonly used: ICI monotherapy or a combination of ICI with chemotherapy (chemo-ICI), depending on the level of PD-L1 expression and the patient’s clinical features (eg, age).^34^ Subgroup analysis from the KEYNOTE042 trial^35^ revealed that treatment-naive patients with PD-L1 (TPS) ranging from 1% to 49% had a response rate of 17% with pembrolizumab monotherapy. Moreover, PD-L1−negative patients also showed potential benefits from nivolumab, achieving a higher median OS compared to chemotherapy.^36^ In our overall dataset, we observed a response rate of 12% for the PD-L1 less than 1% subset, and 18% for the PD-L1 of 1% to 49% subset (eFigure 22 in Supplement 1).

#### PD-L1 TPS of 50% or Greater

In the subgroup of patients with a PD-L1 (TPS) of 50% or greater, the Deep-IO high category provided superior stratification, achieving a 47% response rate compared to a 41% response rate in the PD-L1 high category within the validation cohort. The combined model of high Deep-IO plus high PD-L1 had a response rate of 51% (Figure 3D). This suggests that the complementary use of PD-L1 and Deep-IO could enhance the accuracy of predicting ICI treatment responses more effectively than using either biomarker alone.

#### PD-L1 TPS 1% to 49%

This subgroup of patients with NSCLC is currently treated with a combination of chemotherapy and ICI therapy, in the absence of *EGFR* or *ALK* alterations. Within this patient subgroup, Deep-IO demonstrated strong performance, with an AUC of 0.74 for differentiating ORR and an HR of 0.51 for PFS (eFigure 18 in Supplement 1). Although this was a subgroup analysis involving 61 patients, the performance suggests that Deep-IO could identify those patients who were likely to respond to ICI therapy alone, thereby simplifying their treatment regimen and reducing toxic effects. Overall, we suggest that combining Deep-IO analysis with other standard biomarkers will enhance clinical decision-making, enabling a higher level of precision.^37^

### Limitations

Our study is a hypothesis-generating proof of principle investigation, and as such, it had limitations. First, the data on PD-L1 and TMB were not uniformly available across all samples, which could introduce bias with regards to real performance of these factors compared to Deep-IO (eFigure 3 in Supplement 1). Second, the developed pipeline is not entirely automated as it requires pathologist assessment of the region of interest on each slide. While this step is currently essential, developing automated methods could enhance reproducibility and scalability. Third, while our model demonstrated association with ICI response, it fell short of the ideal AUC threshold of more than 0.8. Further refinement, such as using vision transformers or multimodal models, could potentially improve its accuracy. Fourth, our research focused solely on ICI monotherapy, despite the existence of multiple approved first-line treatments that combine chemotherapy (chemo-ICI). Further research could explore artificial intelligence−based analysis to identify features that correspond with response to chemo-ICI therapy; as well as to assist in determining which patients are likely to benefit from adjuvant or neoadjuvant ICI or chemo-ICI treatment in early-stage NSCLC.^38,39^

## Conclusions

In conclusion, the deep learning model has the capability to predict ICI responses directly from a single image of an H&E-stained slide. This analysis could serve as an auxiliary biomarker alongside PD-L1 immunohistochemistry for advanced NSCLC, potentially enhancing patient stratification and improving selection of tailored therapy for each patient while optimizing the benefit-cost balance in ICI treatment. Further validation of the clinical utility of Deep-IO or a similar method for predicting response to various treatment regimens in NSCLC will be of interest.

## Notes

Supplement 1. eMethods eReferences eFigure 1. Deep-IO pipeline eFigure 2. Inception_v3 architecture eFigure 3. Biomarker data eFigure 4. QC for image tile analysis eFigure 5. Image tile distribution eFigure 6. Objective Response Rate (ORR) eFigure 7. Correlation between image tile count and Deep-IO score eFigure 8. Deep-IO model performance eFigure 9. Confusion matrix on ICI response classification eFigure 10. Model interpretability eFigure 11. Deep-IO and clinical outcomes in validation subsets eFigure 12. Response rate and treatment line eFigure 13. Deep-IO and treatment line eFigure 14. Deep-IO and ICI agent eFigure 15. Deep-IO and histology eFigure 16. Univariate analysis of PFS eFigure 17. Univariate analysis of OS eFigure 18. Deep-IO vs TILs and ICI outcomes by PD-L1 subgroups eFigure 19. Deep-IO vs other known biomarkers eFigure 20. Deep-IO vs PD-L1 and ICI outcome eFigure 21. ORR and performance power in validation subsets eFigure 22. Response rate in overall material

Supplement 2. Data Sharing Statement
