# Supplementary material for: Deep Learning Model for Predicting Immunotherapy Response in Advanced Non−Small Cell Lung Cancer
Source: JAMA Oncol. 2024 Dec 26;11(2):109–18. doi: 10.1001/jamaoncol.2024.5356 (PMC11843371; doi:10.1001/jamaoncol.2024.5356)
Supplement: Supplement 1. — eMethods eReferences eFigure 1. Deep-IO pipeline eFigure 2. Inception_v3 architecture eFigure 3. Biomarker data eFigure 4. QC for image tile analysis eFigure 5. Image tile distribution eFigure 6. Objective Response Rate (ORR) eFigure 7. Correlation between image tile count and Deep-IO score eFigure 8. Deep-IO model performance eFigure 9. Confusion matrix on ICI response classification eFigure 10. Model interpretability eFigure 11. Deep-IO and clinical outcomes in validation subsets eFigure 12. Response rate and treatment line eFigure 13. Deep-IO and treatment line eFigure 14. Deep-IO and ICI agent eFigure 15. Deep-IO and histology eFigure 16. Univariate analysis of PFS eFigure 17. Univariate analysis of OS eFigure 18. Deep-IO vs TILs and ICI outcomes by PD-L1 subgroups eFigure 19. Deep-IO vs other known biomarkers eFigure 20. Deep-IO vs PD-L1 and ICI outcome eFigure 21. ORR and performance power in validation subsets eFigure 22. Response rate in overall material [file jamaoncol-e245356-s001.pdf]

Supplemental Online Content

Rakaee M, Tafavvoghi M, Ricciuti B, et al. Deep Learning Model for Predicting Immunotherapy Response in Advanced Non−Small Cell Lung Cancer. *JAMA Oncol*. Published online December 26, 2024. doi:10.1001/jamaoncol.2024.5356

eMethods

eReferences

eFigure 1. Deep-IO pipeline

eFigure 2. Inception_v3 architecture

eFigure 3. Biomarker data

eFigure 4. QC for image tile analysis

eFigure 5. Image tile distribution

eFigure 6. Objective Response Rate (ORR)

eFigure 7. Correlation between image tile count and Deep-IO score

eFigure 8. Deep-IO model performance

eFigure 9. Confusion matrix on ICI response classification

eFigure 10. Model interpretability

eFigure 11. Deep-IO and clinical outcomes in validation subsets

eFigure 12. Response rate and treatment line

eFigure 13. Deep-IO and treatment line

eFigure 14. Deep-IO and ICI agent

eFigure 15. Deep-IO and histology

eFigure 16. Univariate analysis of PFS

eFigure 17. Univariate analysis of OS

eFigure 18. Deep-IO vs TILs and ICI outcomes by PD-L1 subgroups

eFigure 19. Deep-IO vs other known biomarkers

eFigure 20. Deep-IO vs PD-L1 and ICI outcome

eFigure 21. ORR and performance power in validation subsets

eFigure 22. Response rate in overall material

This supplemental material has been provided by the authors to give readers additional information about their work.

© 2024 Rakaee et al. *JAMA Oncol.*

Supplementary eMethods

Ethical clearance

Experienced oncologists, blinded to the computational analysis of the histology images, collected clinicopathological data and clinical outcomes from individual patient records. The institutional review boards (IRB) at each center granted approval for this study: Dana Farber Cancer Institute (DFCI): DF/HCC #02-180, 11-104, 13-364, and/or 17-000; and specific IRB approval for digital pathology slide analysis was obtained for DFCI/ Brigham and Women's Hospital (BWH) with IRB ID: # 2021P000557 ; Fondazione Policlinico Universitario Campus Bio Medico University of Rome (FPUCBM): PAR 45.23 OSS; Amsterdam University Medical Center (AUMC): U2017.003; Imperial College London (ICL):17/WA/0161/R18009. For data privacy, all digital images were anonymized prior to shipping, and all analyses were conducted on local high-power systems rather than cloud services. No sensitive data, including medical record number, race, ethnic origin, health status, or disabilities were shared.

A. Datasets

Inclusion and exclusion criteria

Developmental cohort: The DFCI cohort included a consecutive collection of 751 patients with histologically or cytologically confirmed NSCLC who underwent targeted next-generation sequencing (NGS) through OncoPanel and were treated with immune checkpoint inhibitors (ICI) monotherapy. Validation cohorts: Patients were consecutively selected based on histologically confirmed NSCLC who underwent ICI monotherapy.

Exclusions were as follows:

Criteria	DFCI	FPUCBM	AUMC	ICL
**Initial patients**	751	145	161	78
**Cytology samples**	128	N/A	N/A	N/A
**No tumor tissue**	3	1	N/A	1
**Poor image quality**	6	7	8	N/A
**Incomplete outcome data (ORR)**	N/A	N/A	23	N/A
**Final cases**	614	137	130	77

© 2024 Rakaee et al. JAMA Oncol.

Digitalization of histology slides

For the DFCI developmental cohort, Hematoxylin and Eosin (H&E)-stained slides were digitized at resolution of 0.49 µm/px using Aperio ScanScope AT (svs format, Leica Biosystems, Germany). The validation cohort slides were scanned at different resolutions: the ICL and AUMC cohorts at 0.24 µm/px (mrxs format, 3DHistech, Hungary) and the FPUCBM cohort at 0.23 µm/px (ndpi format, NanoZoomer, Hamamatsu, Japan).

Tumor delineation

In the developmental cohort, all patients had Oncopanel Next-Generation Sequencing (NGS). Pathologists marked regions on H&E-stained slides that were rich in tumor cells and free from artifacts. These areas were then harvested from matching unstained slides for NGS [1]. For NGS purpose, a minimum tumor cellularity of >20% is required. The same annotated regions were utilized for this study and digital image analysis. In lymph node (LN) tissues, existing lymphoid stroma was excluded, focusing only on the tumor nests and desmoplastic stromal areas as identified by the pathologist, following the guidelines of the International Immuno-Oncology Working Group [2]. For the validation cohort, only a subset of the cases was pre-annotated. The remaining cases were annotated by thoracic pathologists (LTB, ER) using the aforementioned criteria.

B. Pre-processing

Image tiling

The whole-slide images (WSI) are typically very large images that cannot be fed to the deep learning model at once. Therefore, tiling is often used as a pre-processing step to break down these large images into smaller tiles. Considering the variation in resolutions of WSI derived from different scanners, for harmonization purposes, all WSI underwent a down-sampling process using Qupath (v.0.4.3) [3]. The delineated tumor regions were tessellated and divided into image tiles with a size of 512x512 pixels and a spatial resolution of 0.50 µm /px. Additionally, the tiles were generated with a minimal overlap of 32 µm to ensure that features within each tile are effectively preserved and to increase the number of representative tumor tiles for the training.

© 2024 Rakaee et al. JAMA Oncol.

Tile QC

The image tiles may contain a significant amount of white background with minimum tissue regions and/or pen markers drawn by pathologists during diagnoses. Including tiles with white background or pen markers in the training set could negatively impact model performance.

To exclude the tiles with minimal tissue content, an automated procedure was built. First, the image tile was converted to grayscale, allowing to differentiate between tissue and background. Then, an adaptive thresholding technique was applied to segment the tissue from the background [4]. To refine the segmentation, morphological closing and opening operations were performed. These operations allow to remove small objects and bridge gaps within the tissue regions. The final step involved calculating the proportion of tissue within each tile. This was performed by comparing the number of pixels identified as tissue to the total number of pixels in the tile. Tiles with a tissue content below a predefined threshold (<50%) [5] were excluded from subsequent analyses.

To exclude tiles with pen markers, an automated procedure was built. After reading each image tile, the RGB components were separated to individually analyze the red, green, and blue channels. Then, the percentage of each color channel in the image tile was calculated to identify the presence of specific marker signs [6]. The excluded/included tiles per patients are randomly checked by pathologists.

Color normalization

Differences in scanners and staining processes can cause significant color variations in WSIs. Therefore, it is essential to address these variations using a color normalization technique. The color normalization technique utilized in our study is based on the approach proposed by Macenko [7], which is widely used for histopathology images [8,9]. Briefly, for a given image tile, the color values are transformed into a format that quantifies the intensity of staining, known as Optical Density (OD). After this conversion, the OD values are decomposed into two vectors: a stain vector and a saturation vector. Then, using a set of reference image tiles, the stain vector is computed by applying Singular Value Decomposition (SVD) technique. The stain vector obtained from SVD is then utilized to normalize the OD values of the input image tile and then generate the final normalized image tile.

© 2024 Rakaee et al. JAMA Oncol.

Data balancing

In the developmental cohort, the class distribution was imbalance consisting of 26% responders and 74% non-responders. To prevent the model from being biased towards the majority class and to ensure that it learns from sufficient samples of both classes, the data imbalance in the training set was addressed by oversampling the minority class [10,11]. Instances of the minority class were replicated three times using random augmentation techniques. The augmentation included flipping and 90-degree rotations.

C. Deep-IO Model

Model training

The model was trained on the Objective Response Rate (ORR; according to RECIST) for immune checkpoint inhibitor treatment. In this classification, patients with complete or partial responses were labeled as "responders", while those with progressive or stable disease were labeled as "non-responders". The developmental cohort was randomly divided at a patient level for each class into an 85% training set (521 patients; 275,078 tiles, including oversampling) and a 15% testing set (93 patients; 36,254 tiles).

The training was performed using Inception_v3 convolutional neural network (CNN) with pre-trained weights in PyTorch (v.2.0.1) [12,13] . The balanced and color normalized tiles (size 512x512 pixels) were fed into the model configured with the Adam optimizer [14], using following hyperparameters: batch size of 64, learning rate of 5e-06, and dropout rate of 0.33. A combination of different hyperparameters and CNN (ResNet18, ResNet50, ResNet110, EfficientNet_v2, ShuffleNet_v2, RegNet; https://pytorch.org/vision/stable/models.html) models were examined to achieve the model optimal performance.

The model primarily utilized histological features from the H&E-stained images. Features such as cellular morphology, tissue architecture, and patterns indicative of immune response were implicitly learned by the deep learning model during training. Hyperparameter tuning was performed manually by testing different combinations of parameters. This included adjusting learning rates (e.g., 1e-5, 5e-6), batch sizes (e.g., 32, 64, 128; based on CNN model and GPU capacity), and dropout rates (e.g., 0.22, 0.33). Each combination was closely

monitored via TensorBoard (PyTorch) focusing on cross-entropy loss [15]. Training was ended either when there was no improvement in the test set loss for 50 consecutive epochs or upon signs of overfitting. PyTorch built-in functions, including weight decay, batch normalization and augmentation were employed for training and regularization to enhance model robustness. The model training process was carried out on NVIDIA RTX4090 graphical processing unit (GPU). The hardware specifications included GPU with 24 GB of memory (CUDA version 12.2), running on an UBUNTU (v. 20.4) OS, 32 core-CPU and 128 GB of RAM.

D. Performance Evaluation

Internal model performance

The prediction probability (PP) for each tile, derived from the softmax function (**eFigure 2**), ranged from 0 to 1, with PP scores < 0.5 classified as class 0 (non-responder) and those ≥ 0.5 as class 1 (responder). For patient-level predictions, the scores of individual tiles were averaged to derive a final score, referred to as the Deep-IO score, using the same classification threshold. To assess the final model's performance on the test set, various metrics were computed including the F1 score, sensitivity, specificity, precision, recall, and the area under the receiver operating characteristic (AUC) curve. The confidence interval for the AUC is estimated by performing 1,000 bootstrap re-samplings. Additionally, a confusion matrix was used to provide a detailed analysis of the model accuracy.

External validation

To evaluate the generalizability of the Deep-IO model, the final and fine-tuned weights (obtained from the test set with minimal loss) were applied to an external validation cohort. This cohort consisted of 344 patients (138,612 tiles), processed using the same preprocessing procedures as the developmental cohort. The same cutoff used in the test set was applied for classifying responders (scores ≥ 0.5) and non-responders (scores < 0.5) in the evaluation metrics.

The model training and test optimization were conducted by two investigators (F.J, M.T) blinded to the external validation dataset.

© 2024 Rakaee et al. JAMA Oncol.

Model visualization

To address the interpretability of Deep-IO model, we generated the visualization of our model outcome on the images using Gradient-weighted Class Activation Mapping (Grad-CAM) [16]. The resulting heatmaps highlighted the most indicative region of the images based on the region's influence on the model prediction outcome both at the tile and WSI level. Following the training of our model, and achieving the final optimal weight, we employed these weights to generate Grad-CAM visualizations utilizing the last convolutional layer within our model.

To objectively assess the model's focus across key histological compartments (tumor, stroma, inflammatory) in WSIs, we developed a semi-quantitative scoring system based on the intensity of red-colored Grad-CAM overlays. Tiles adjusted for Grad-CAM overlays, corresponding to each class (responder and non-responder), were stitched together to create a comprehensive slide overview. This process was applied to randomly selected sets of 25 patients from each class prediction. Scoring is assigned as follows: 1 (none/low focus): A minimal presence of red overlays, indicating low model attention. 2 (moderate focus): A moderate presence of red overlays, suggesting noticeable model attention. 3 (high focus): An extensive presence of red overlays, denoting high model focus.

The assessment was performed by an experienced thoracic pathologist (L.T.B), and scores were recorded for each patient based on the predominant focus observed in each compartment: Tumor: areas containing malignant epithelial cells. Stroma: connective tissue and the extracellular matrix around tumor cells within the tumor bed or core. Inflammatory reaction area: regions characterized by highly diffused inflammatory cell infiltration or the presence of immune cell aggregates, if they exist within the WSI.

Mutation and TMB analysis

In the development cohort, DNA analysis was conducted using the OncoPanel Next-Generation Sequencing (NGS) panel. Throughout the process, three progressive versions of OncoPanel were employed, encompassing 275, 300, and 447 genes associated with cancer, analyzed through a thoroughly validated in-house computational pipeline [17]. Tumor Mutational Burden (TMB) was characterized as the count of non-synonymous missense mutations and small indel variants per megabase of the sequenced genome. Within the

© 2024 Rakaee et al. JAMA Oncol.

FPUCBM validation subgroup, TMB calculations were performed on subset of patients using the FoundationOne CDx NGS panel. For mutation analysis in the ICL and AUMC sub-cohorts, QIAseq targeted DNA panel was used, with multiplex PCR targeted enrichment technology for ICL, and the TSACP/MiSeq Illumina platform for AUMC.

PD-L1 expression

The PD-L1 Tumor Proportion Score (TPS) was calculated based on the percentage of tumor cells showing PD-L1 positivity in comparison to the total number of tumor epithelial cells on the slide within the targeted area, regardless of the intensity of staining. A minimum of 100 viable tumor cells was necessary to assess PD-L1 expression. PD-L1 data were retrieved from the patient's medical records.

TILs density

Supervised machine learning algorithms were sequentially utilized within the Qupath environment to methodically develop a scoring system for TILs (tumor-infiltrating lymphocytes) [18]. The process was structured as follows: Initially, color deconvolution techniques were used to determine the stain vectors and to equalize the RGB channels for each slide, addressing variations in H&E stain intensity across slides. Next, watershed segmentation was employed to distinguish cells by considering their size, shape, and the optical density of nuclei in the hematoxylin layer, which involved computing 33 distinct features for each cell. This was followed by the incorporation of intensity and smoothed object attributes, along with the calculation of Haralick texture features and gaussian-weighted averages for each cell. Lastly, cell profiling was carried out. Here, a classifier based on the random forest algorithm was developed to categorize cells as TILs, tumor cells, or stroma cells. TILs were specifically identified as mononuclear immune cells, including lymphocytes and plasma cells.

Endpoints & additional statistics

Deep-IO probability scores were categorized using median and tertile cutoffs from the validation cohort. For comparing continuous variables, the Mann–Whitney U test was used for two groups. Spearman correlation was used for two continuous variables. The chi-square test was employed to evaluate the association between two categorical variables. ORR was

© 2024 Rakaee et al. JAMA Oncol.

collected based on RECIST v.1.1 criteria. Progression-Free Survival (PFS) calculated from the onset of ICI therapy to disease progression or death, and Overall Survival (OS) from the start of ICI treatment to death or the last follow-up. Patients without events at their last assessment were censored. Using the Kaplan-Meier method, we estimated event-time distributions and employed log-rank tests to assess the differences. Hazard ratios were derived from univariate and multivariable Cox models, with their proportional hazard assumptions verified using Schoenfeld residuals.

AUC is calculated by ROC analysis to determine the sensitivity, specificity, positive predictive value (PPV), and negative predictive value (NPV) of continuous variables (Deep-IO, TMB, PD-L1, TILs). The Deep-IO and PD-L1 variables were combined through a logistic regression model to calculate a weighted sum. Survival and statistical analyses were conducted using R and Python relevant packages considering P-values below 0.05 as statistically significant.

Extended references

1. Sholl, L. M. et al. Institutional implementation of clinical tumor profiling on an unselected cancer population. JCI Insight 1, (2016).
2. Hendry, S. et al. Assessing Tumor-Infiltrating Lymphocytes in Solid Tumors: A Practical Review for Pathologists and Proposal for a Standardized Method from the International Immuno-Oncology Biomarkers Working Group: Part 2: TILs in Melanoma, Gastrointestinal Tract Carcinom. Advances in Anatomic Pathology vol. 24 311–335 Preprint at https://doi.org/10.1097/PAP.0000000000000161 (2017).
3. Bankhead, P. et al. QuPath: Open source software for digital pathology image analysis. Sci Rep 7, 1–7 (2017).
4. Roy, P. et al. Adaptive thresholding: A comparative study. 2014 International Conference on Control, Instrumentation, Communication and Computational Technologies, ICCICCT 2014 1182–1186 (2014) doi:10.1109/ICCICCT.2014.6993140.
5. Wang, Y. et al. Improved breast cancer histological grading using deep learning. Annals of Oncology 33, 89–98 (2022).
6. Papadopoulos, K. M. et al. Overall Survival Time Estimation for Epithelioid Peritoneal Mesothelioma Patients from Whole-Slide Images. BioMedInformatics 2024, Vol. 4, Pages 823-836 4, 823–836 (2024).
7. Macenko, M. et al. A method for normalizing histology slides for quantitative analysis. Proceedings - 2009 IEEE International Symposium on Biomedical Imaging: From Nano to Macro, ISBI 2009 1107–1110 (2009) doi:10.1109/ISBI.2009.5193250.
8. Klauschen, F. et al. Toward Explainable Artificial Intelligence for Precision Pathology. Annual Review of Pathology: Mechanisms of Disease 19, 541–570 (2024).

© 2024 Rakaee et al. JAMA Oncol.

9. Srinidhi, C. L., Ciga, O. & Martel, A. L. Deep neural network models for computational histopathology: A survey. Med Image Anal 67, (2019).
10. Arslan, S. et al. A systematic pan-cancer study on deep learning-based prediction of multi-omic biomarkers from routine pathology images. Communications Medicine 2024 4:1 4, 1–15 (2024).
11. Mohammed, R., Rawashdeh, J. & Abdullah, M. Machine Learning with Oversampling and Undersampling Techniques: Overview Study and Experimental Results. 2020 11th International Conference on Information and Communication Systems, ICICS 2020 243–248 (2020) doi:10.1109/ICICS49469.2020.239556.
12. Coudray, N. et al. Classification and mutation prediction from non–small cell lung cancer histopathology images using deep learning. Nature Medicine 2018 24:10 24, 1559–1567 (2018).
13. Szegedy, C., Vanhoucke, V., Ioffe, S., Shlens, J. & Wojna, Z. Rethinking the Inception Architecture for Computer Vision. Proceedings of the IEEE Computer Society Conference on Computer Vision and Pattern Recognition 2016-December, 2818–2826 (2015).
14. Kingma, D. P. & Ba, J. L. Adam: A Method for Stochastic Optimization. 3rd International Conference on Learning Representations, ICLR 2015 - Conference Track Proceedings (2014).
15. Mao, A., Mohri, M. & Zhong, Y. Cross-Entropy Loss Functions: Theoretical Analysis and Applications. Proc Mach Learn Res 202, 23803–23828 (2023).
16. Selvaraju, R. R. et al. Grad-CAM: Visual Explanations from Deep Networks via Gradient-based Localization. Int J Comput Vis 128, 336–359 (2016).
17. Alessi, J. V et al. SMARCA4 and other SWI/SNF family genomic alterations in non-small cell lung cancer: Clinicopathological characteristics and outcomes to immune checkpoint inhibition. J Thorac Oncol (2021) doi:10.1016/j.jtho.2021.03.024.
18. Rakaee, M. et al. Association of Machine Learning-Based Assessment of Tumor-Infiltrating Lymphocytes on Standard Histologic Images With Outcomes of Immunotherapy in Patients With NSCLC. JAMA Oncol 9, 51–60 (2023).

© 2024 Rakaee et al. JAMA Oncol.

Supplementary eFigures

© 2024 Rakaee et al. JAMA Oncol.

eFigure 1: Deep-IO pipeline. Flowchart illustrating the development of the supervised deep learning model. In the US-based cohort, digital H&E stained slides from biopsy or surgical resection were classified based on the Objective Response Rate to ICI into two groups: responders and non-responders. The dataset was randomly split into training (n= 521) and test (n=93) sets for each class. The digital regions of interest were tiled into smaller images (512x512 pixels). These tiles underwent quality control (QC) to exclude ineligible tiles, followed by color normalization, data balancing, and augmentation. The QC-approved tiles, along with their associated class for each tile, were used as input for training a CNN-based algorithm (see **eFigure 2**). After hyperparameter adjustments, the network generated a probability prediction for each tile input. The tile scores were averaged to determine the patient-level prediction. Performance metrics were evaluated in the test set, and the best-trained model was used for external, independent validation after H&E image resolution harmonization and applying similar preprocessing steps (tiling, tile QC, normalization) as in the developmental set. For more detail see **eMethod**.

© 2024 Rakaee et al. JAMA Oncol.

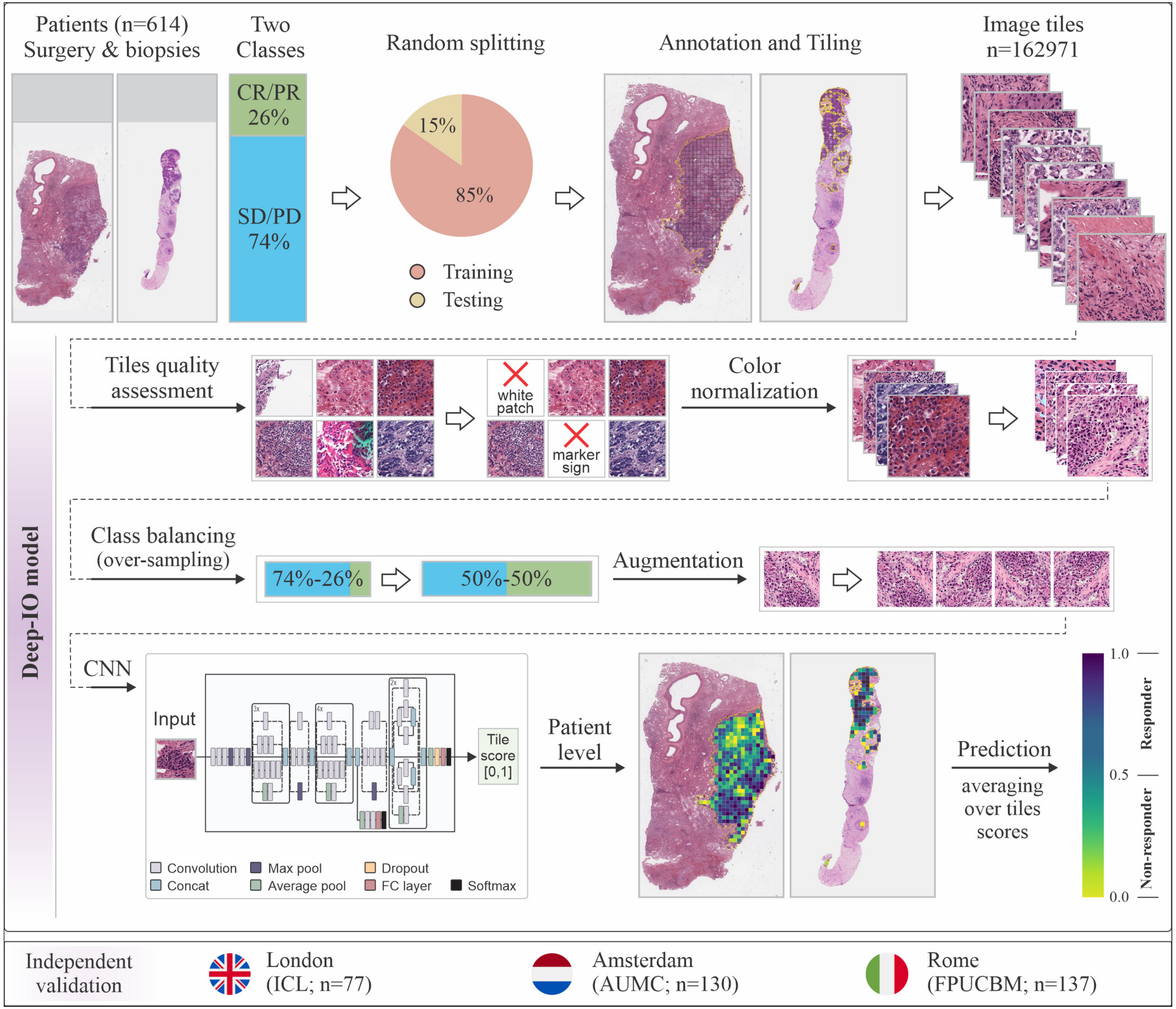

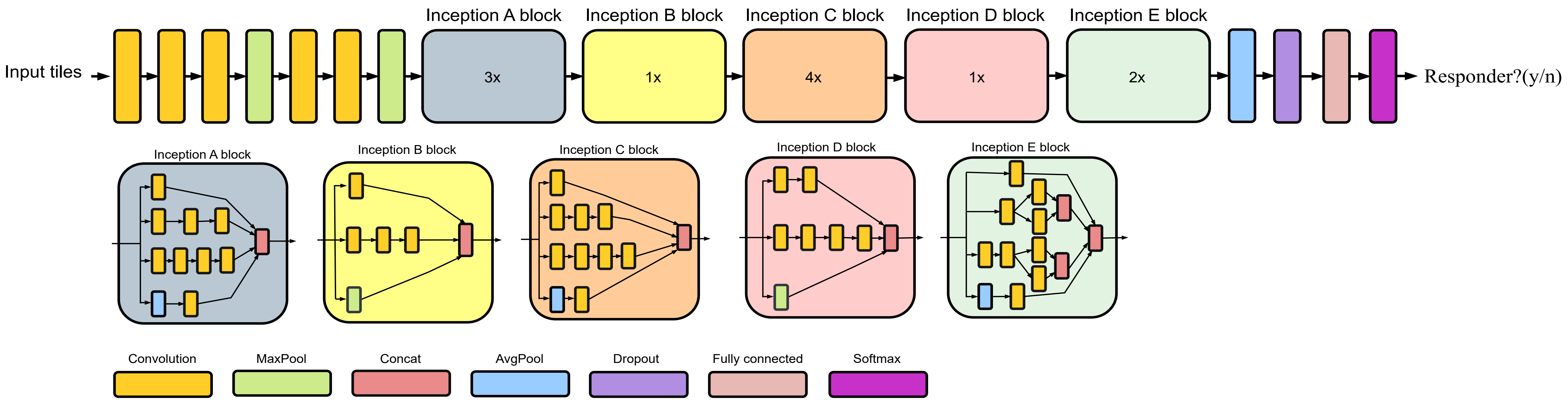

eFigure 2: Inception_v3 architecture: Overview of Inception v3 architecture, representing the flow from input tiles through convolutional blocks (A-E), ending with Softmax layer which outputs binary classes (responder, non-responder). The notation (e.g., 3x) above each block indicates the number of times the block is repeated in the sequence. The model predicts tiles scores in the range of [0,1]. If the score is >= 0.5 the tile is classified as responder, if the score is < 0.5 the tile is classified as non-responder.

© 2024 Rakaee et al. JAMA Oncol.

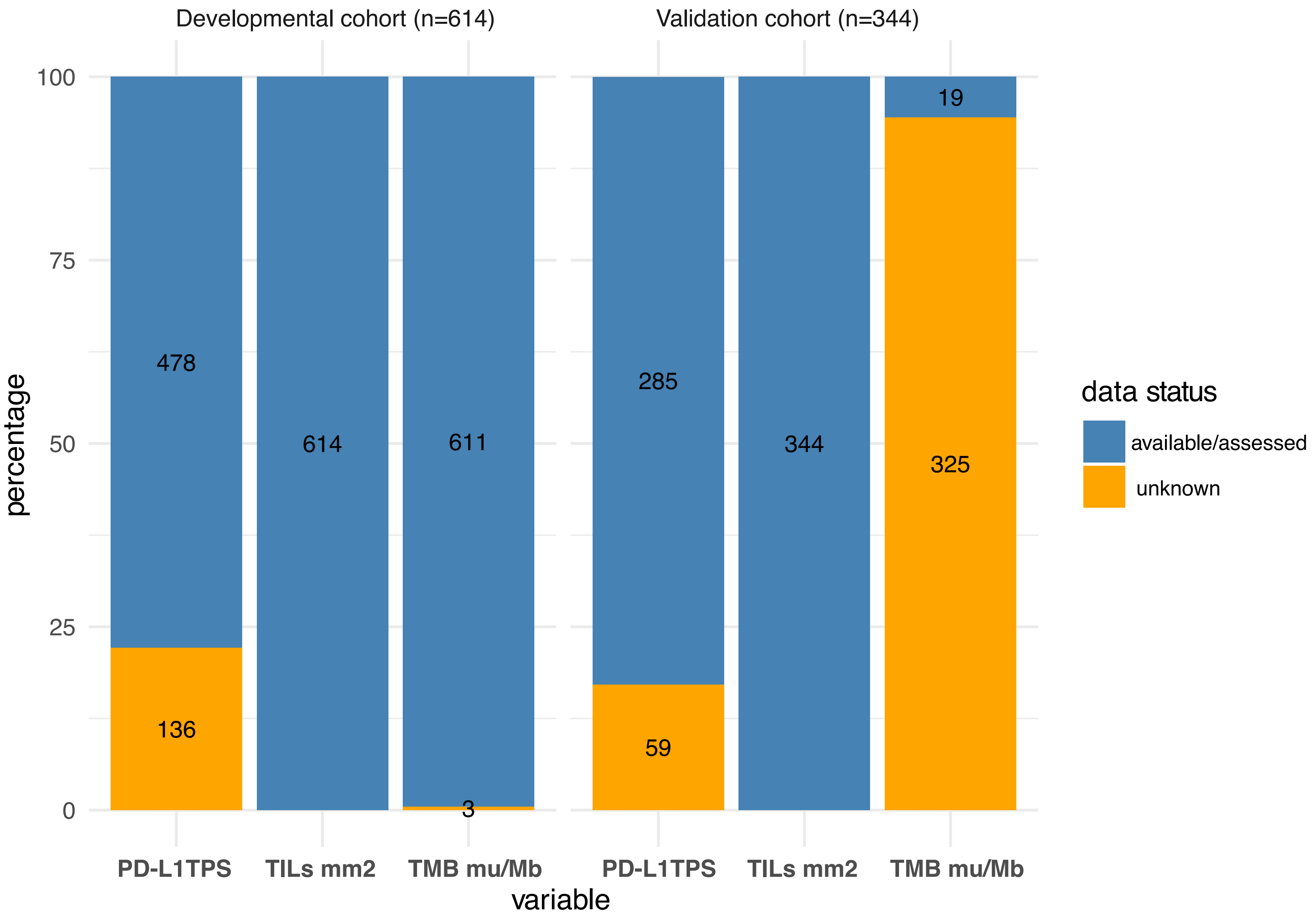

eFigure 3: Biomarker data: Stacked bar plot representing the distribution of unknown and available or processed data for the variables including PD-L1/TPS, TMB mu/Mb, and TILs/mm2 in the developmental and validation cohorts.

© 2024 Rakaee et al. JAMA Oncol.

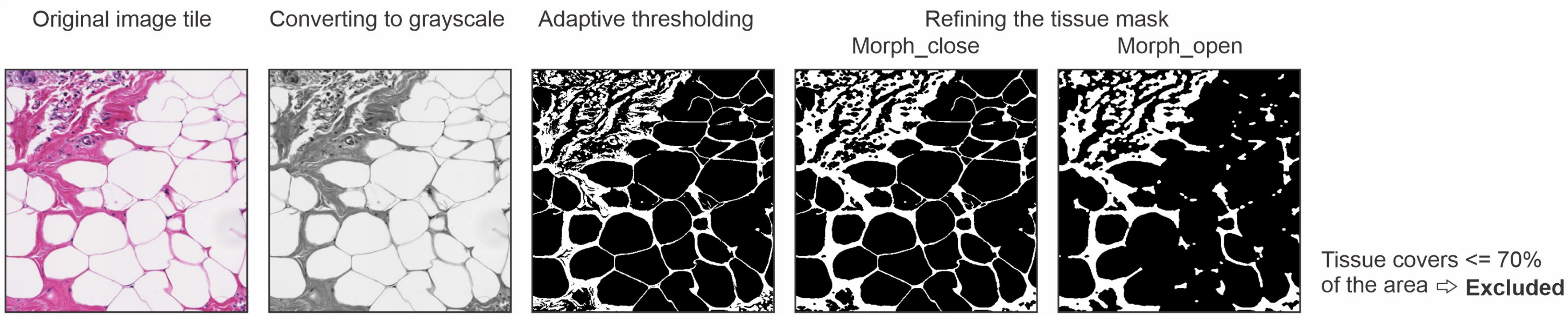

eFigure 4: **QC for image tile analysis**: This panel demonstrates the sequential processing steps applied to a single H&E stained image tile to calculate tissue content. Initially, the image is converted to grayscale to simplify intensity analysis. Subsequently, adaptive thresholding is employed to distinguish tissue regions from the background. The final processing steps involve morphological operations: closing to fill gaps within tissue regions, followed by opening to remove small, isolated artifacts.

© 2024 Rakaee et al. JAMA Oncol

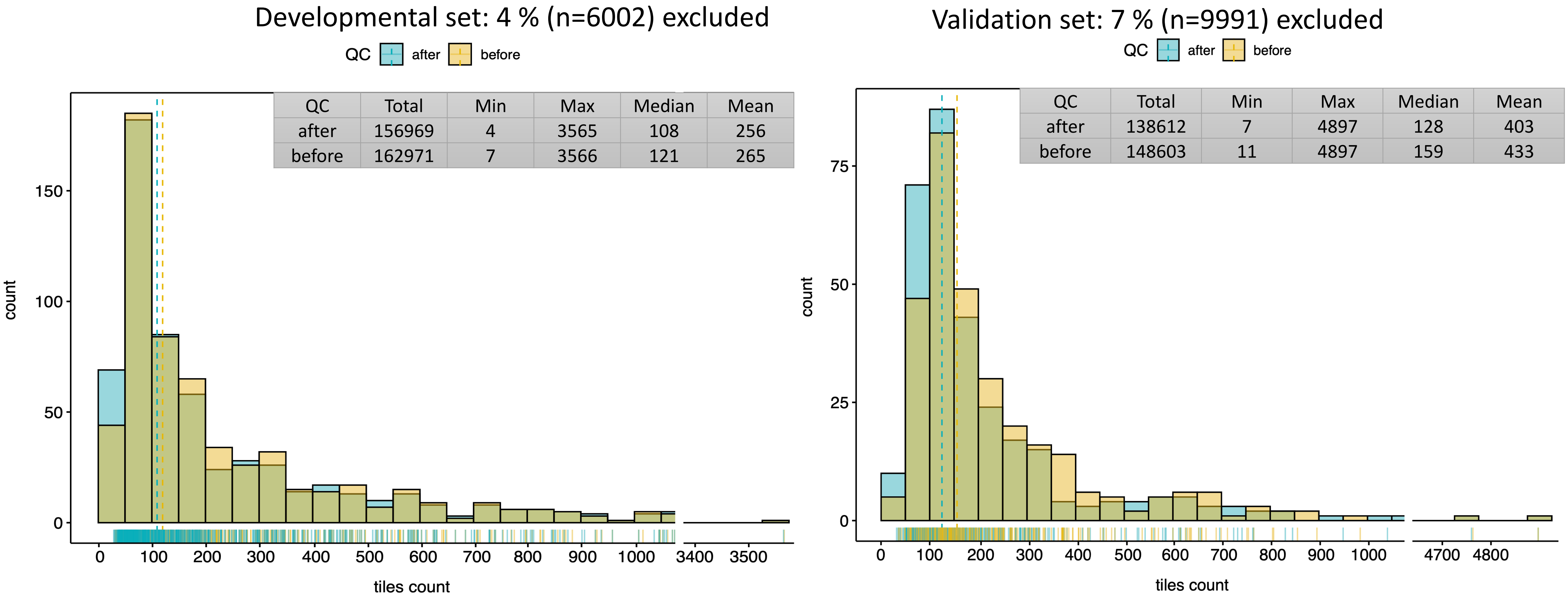

eFigure 5: **Image tile distribution**: Histogram showing the distribution of H&E image tiles per patients before and after QC in developmental and validation cohorts. The x-axis represents the count of image tiles per patients, organized into 50 equal-width bins. The y-axis indicates the frequency of observations within each bin. Dash lines indicate median.

© 2024 Rakaee et al. JAMA Oncol.

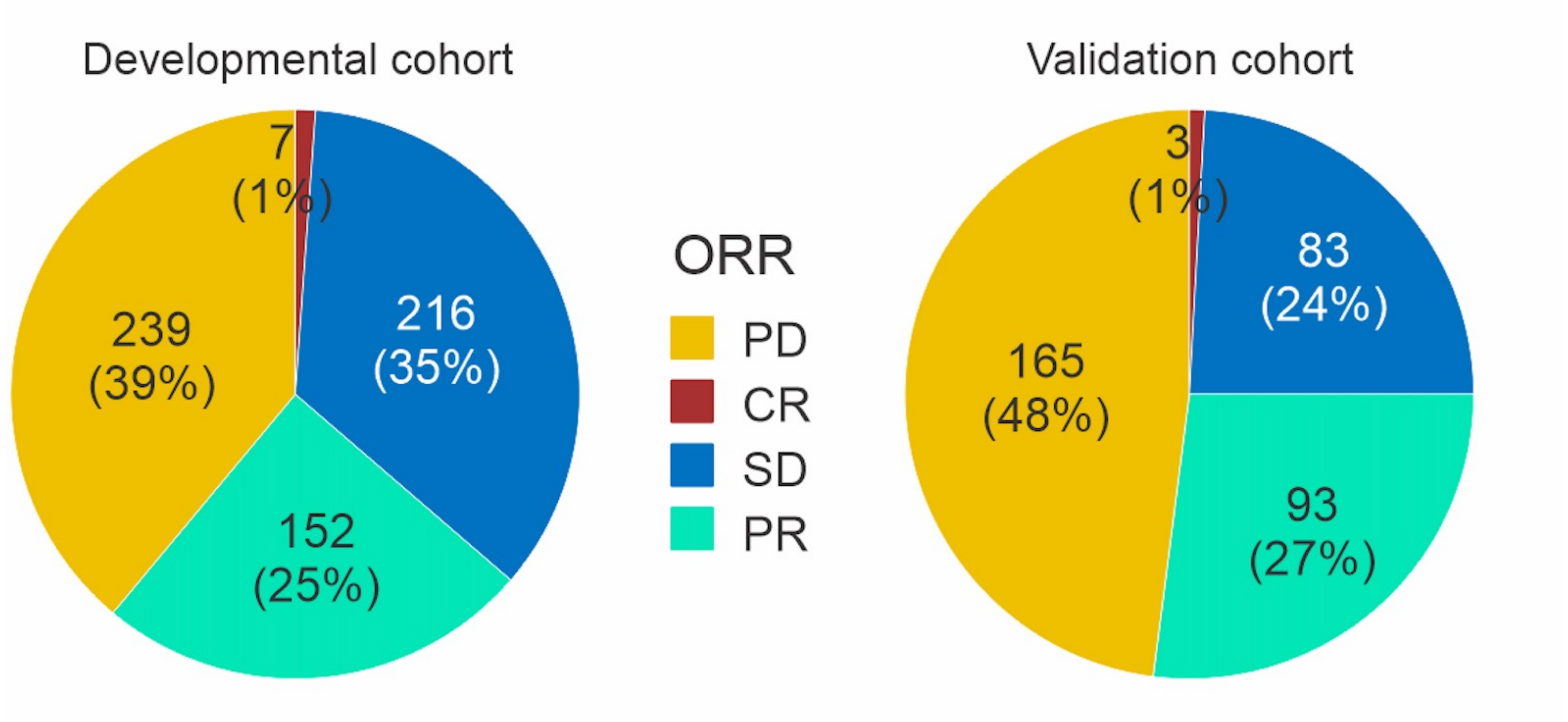

eFigure 6: Objective Response Rate (ORR): Pie charts showing the distribution of objective response rate (CR, complete response; PR, partial response; SD, stable disease; PD, progressive disease) in the developmental and validation cohorts.

© 2024 Rakaee et al. JAMA Oncol

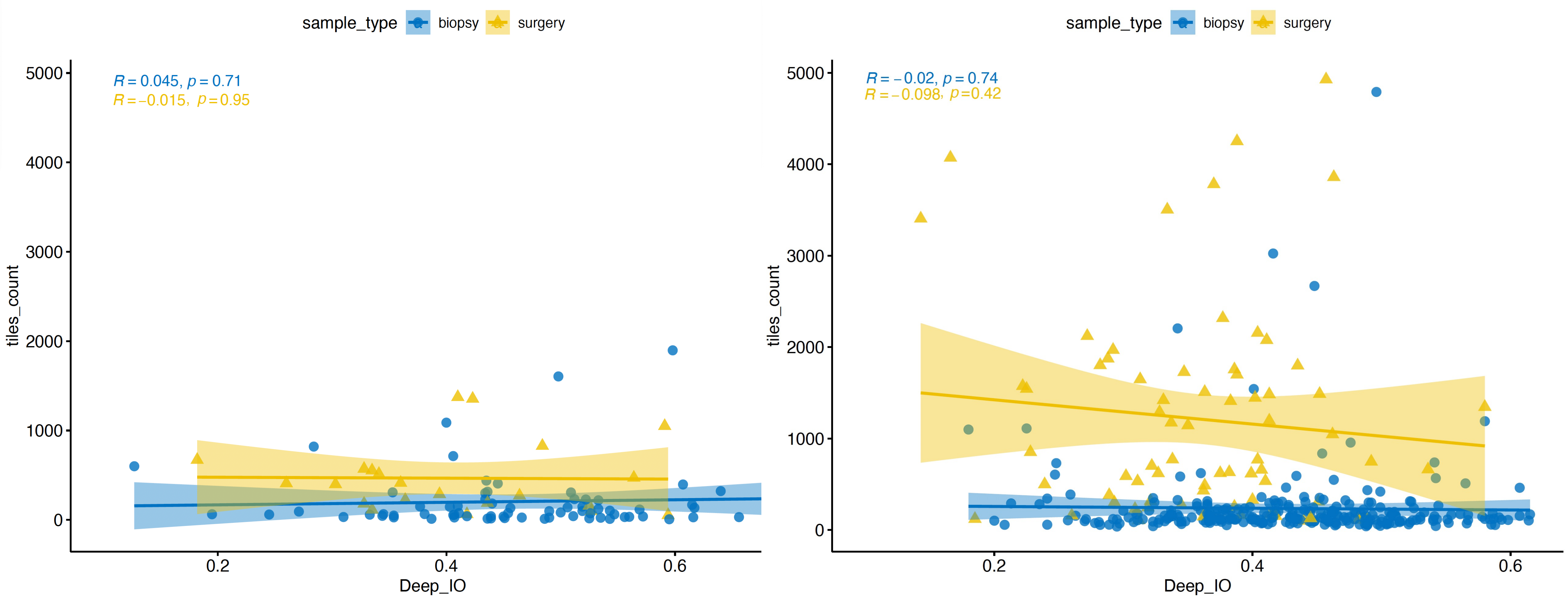

eFigure 7: Correlation between image tile count and Deep-IO score: No correlation is observed between the number of tiles per patient and the patient-level Deep-IO ICI prediction score in biopsy or surgical specimens across both the test (left plot) and external validation (right plot) cohorts.

© 2024 Rakaee et al. JAMA Oncol.

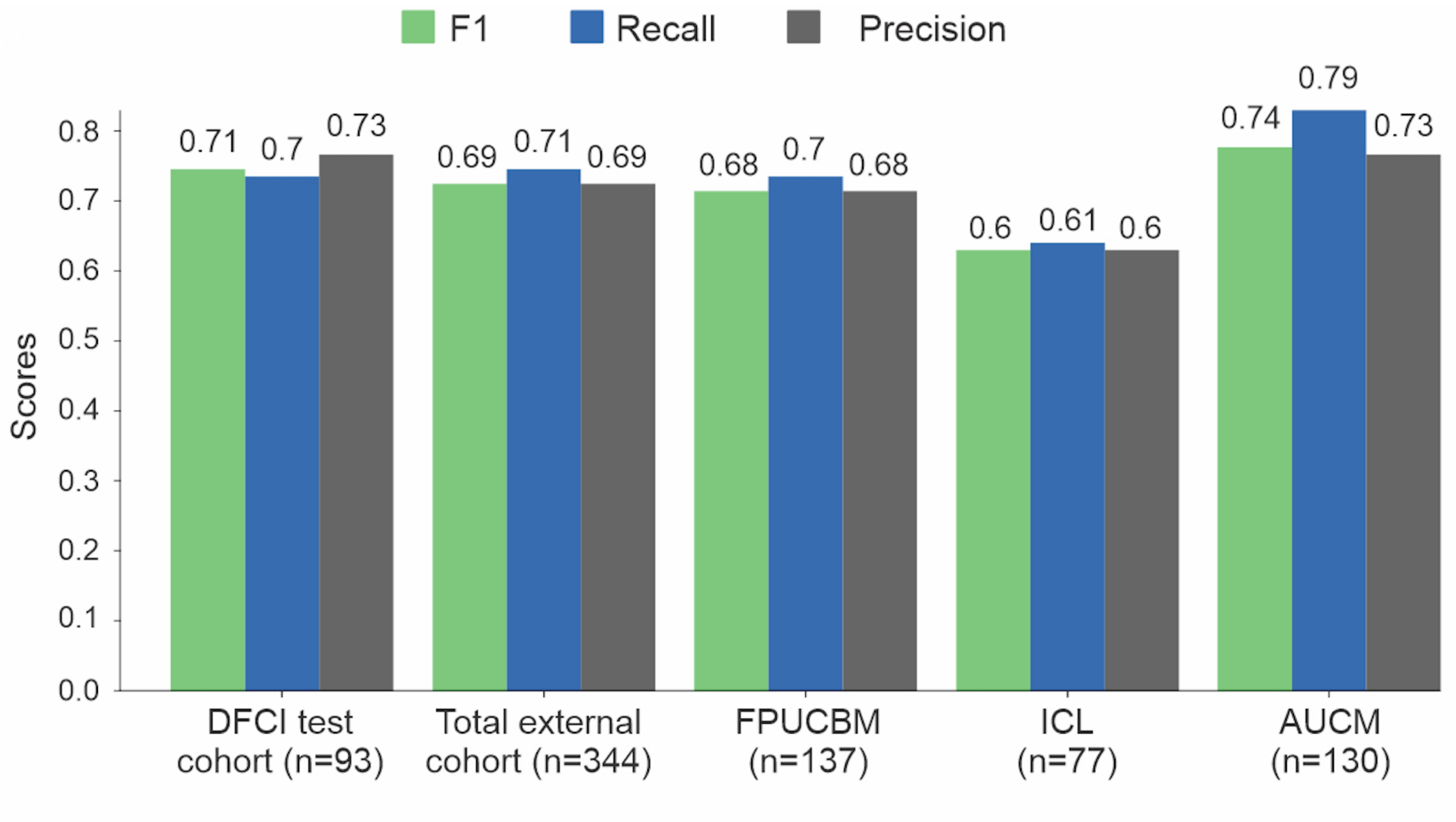

eFigure 8: Deep-IO model performance: Evaluation of the Deep-IO model's performance on the test set, external validation cohort, and three institutions contributing to the validation cohort. The y-axis represents the weighted average of the F1-score, precision, and recall.

© 2024 Rakaee et al. JAMA Oncol.

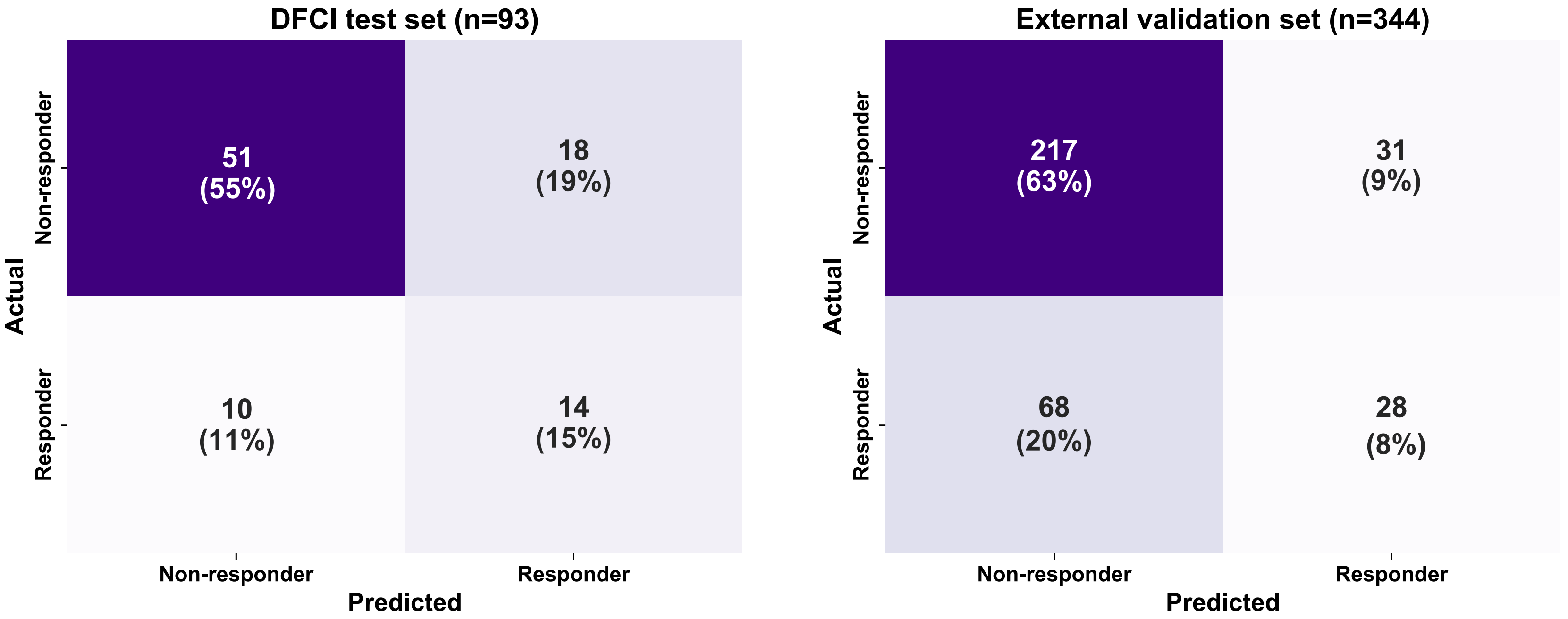

eFigure 9: Confusion matrix on ICI response classification: The confusion matrix illustrates the Deep-IO model's predictive accuracy in classifying patients as responders or non-responders based on objective response rate, showing the predicted classes on the X-axis and the true classes on the Y-axis for the test (left) and external validation (right) cohorts.

© 2024 Rakaee et al. JAMA Oncol.

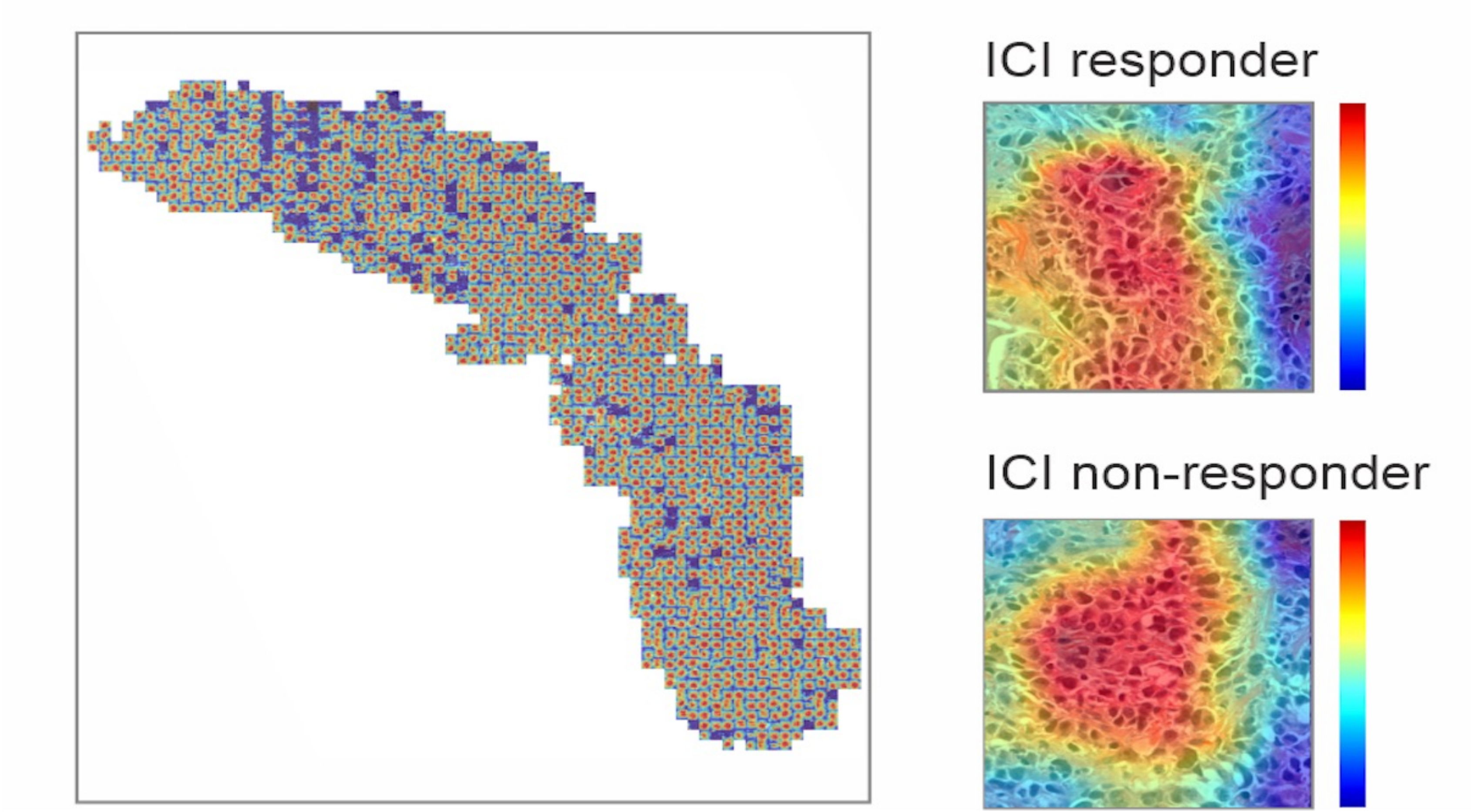

eFigure 10: Model interpretability : Representative images of gradient-weighted class activation mapping (GradCam) visualized at the whole slide image level and on corresponding tile images with GradCam overlays. These overlays are applied to a subset of patients from both responder and non-responder classes. The color hue ranges from red, indicating the highest contribution or importance to the model's decision, to blue, indicating minimal or no influence on the decision for the specified class.

©2024 Rakaee et al. JAMA Oncol

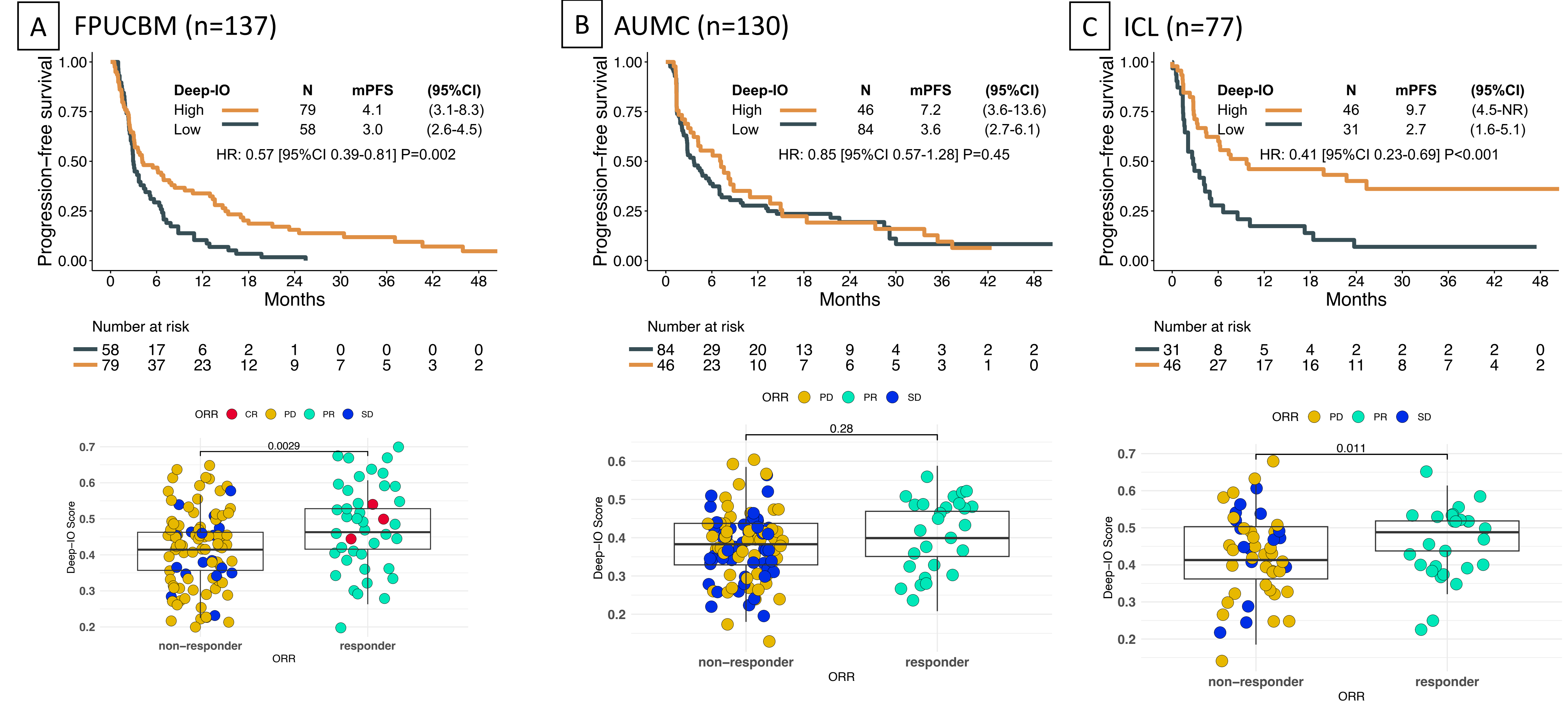

eFigure 11: Deep-IO and clinical outcomes in validation subsets: The upper row features Kaplan-Meier curves for Progression-Free Survival (PFS) in response to ICI within validation subsets: A) FPUCBM (n=137), B) AUMC (n=130), and C) ICL (n=77). The lower row presents the distribution of Deep-IO probability scores across ORR subgroups in the validation subsets, arranged in the same order as above for survival analysis. HR, hazard ratio; CI, confidence interval; mPFS, median PFS in months; mOS, median Overall Survival in months.

© 2024 Rakaee et al. JAMA Oncol.

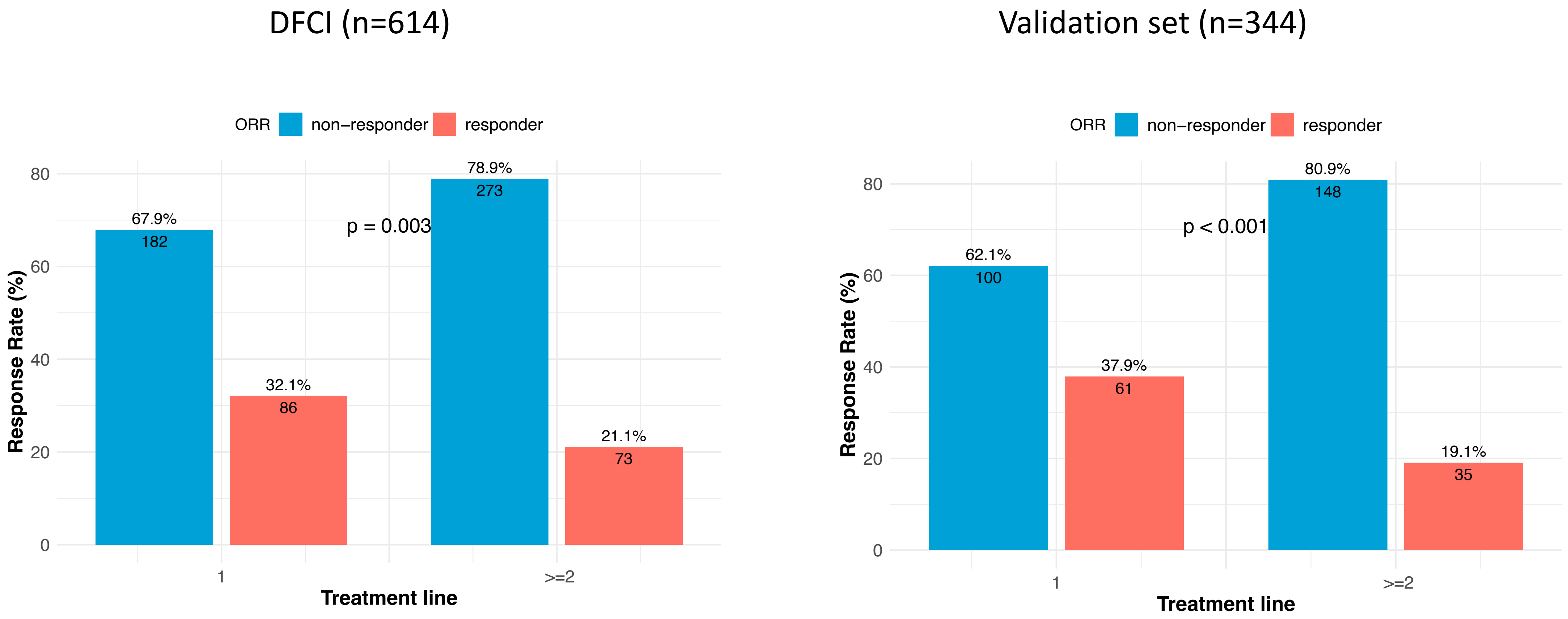

eFigure 12: **Response rate and treatment line:** Objective response rate to ICI across treatment lines in developmental and validation sets.

© 2024 Rakaee et al. JAMA Oncol.

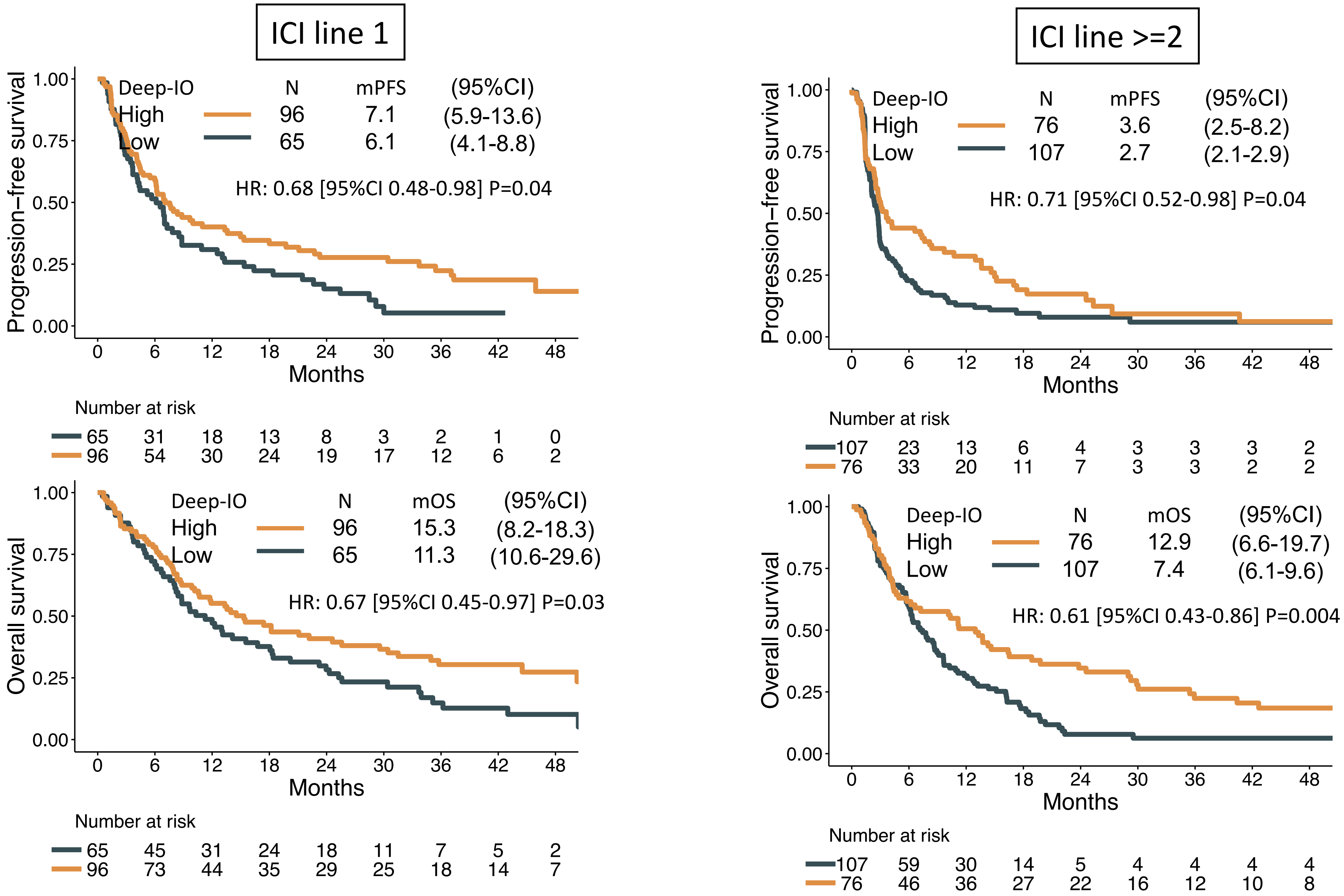

eFigure 13: **Deep-IO and treatment line:** Analysis of progression-free survival (PFS) and overall survival (OS) in treatment subgroups of the validation cohort. HR, hazard ratio; CI, confidence interval; mPFS, median PFS in months; mOS, median OS in months

© 2024 Rakaee et al. JAMA Oncol

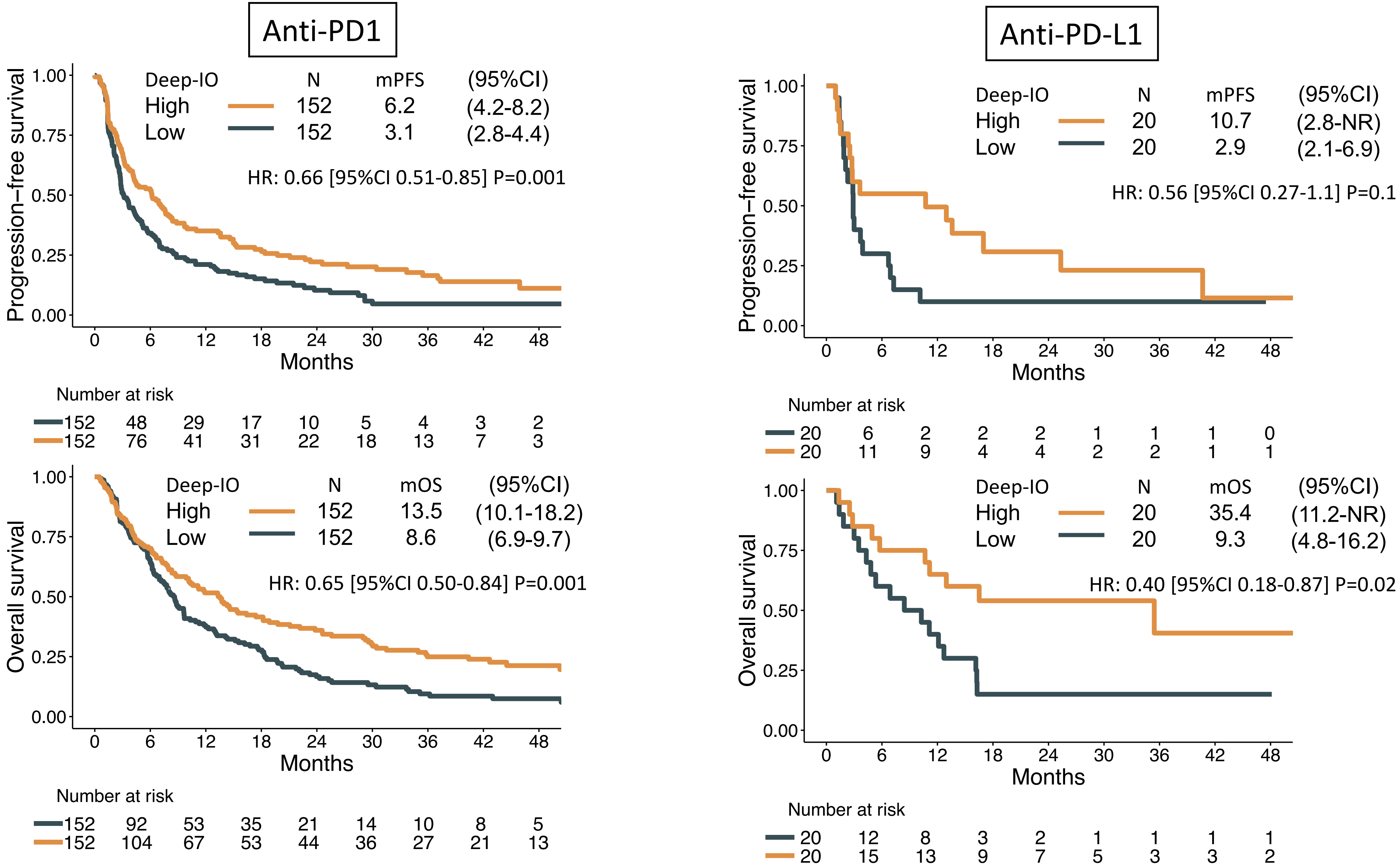

eFigure 14: **Deep-IO and ICI agent:** Analysis of progression-free survival (PFS) and overall survival (OS) in ICI agent subgroups of the validation cohort. HR, hazard ratio; CI, confidence interval; mPFS, median PFS in months; mOS, median OS in months; NR, not reached.

© 2024 Rakaee et al. JAMA Oncol.

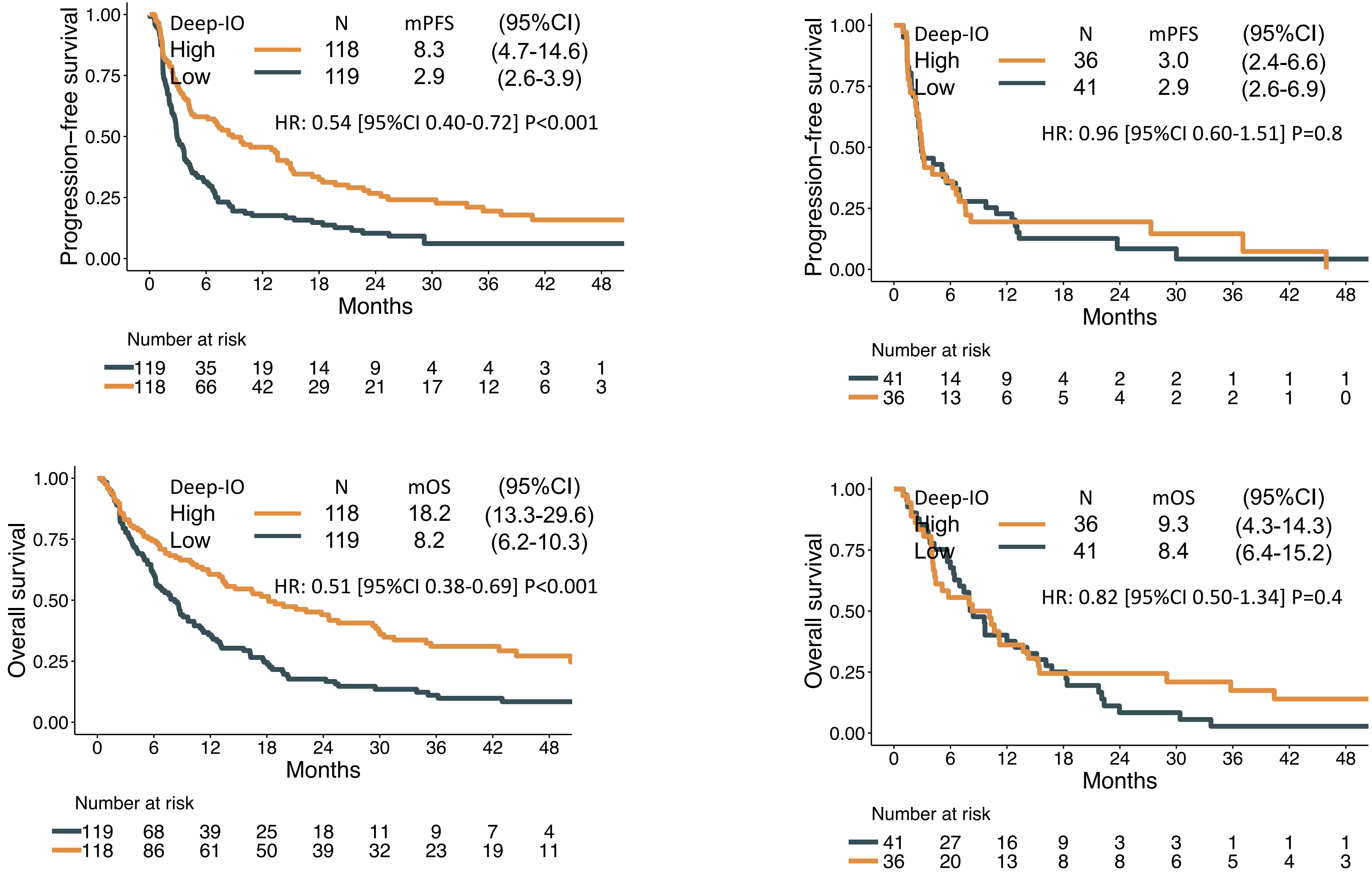

eFigure 15: Deep-IO and histology: Stratified analysis of Deep-IO based on lung adenocarcinoma (n=237, left column) and squamous cell carcinoma (n=77, right column) for PFS and OS analysis in the validation cohort.

© 2024 Rakaee et al. JAMA Oncol.

Abbreviations: HR, hazard ratio; CI, confidence interval; mPFS, median PFS in months; mOS, median OS in months.

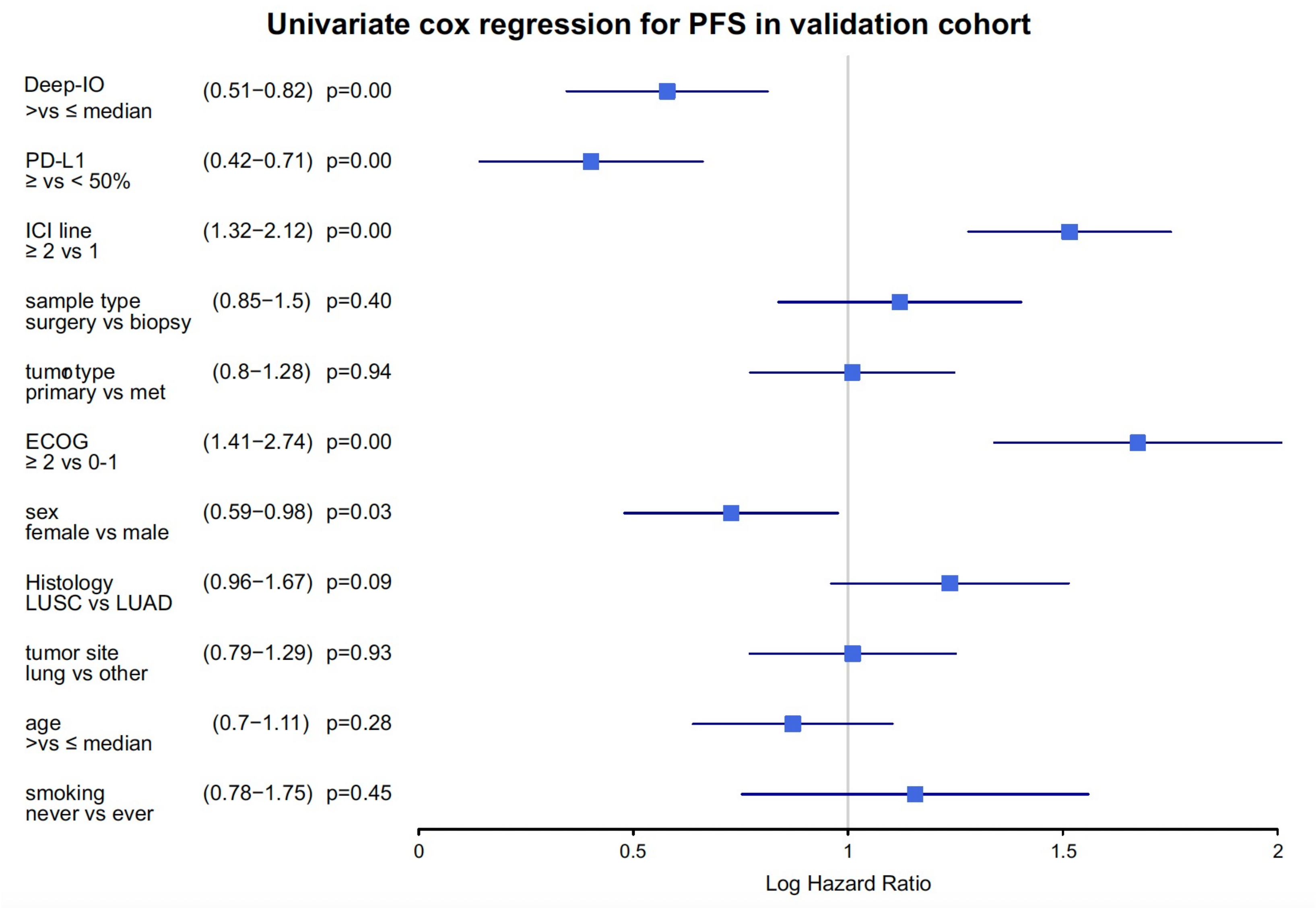

eFigure 16: Univariate analysis of PFS: A forest plot displaying the results of a univariate analysis that identifies variables affecting progression free survival (PFS) to ICI treatment. Each row shows the hazard ratios (HR) and their corresponding 95% confidence intervals with horizontal lines. Number in parenthesis lower and upper 95% confidence intervals.

© 2024 Rakaee et al. JAMA Oncol.

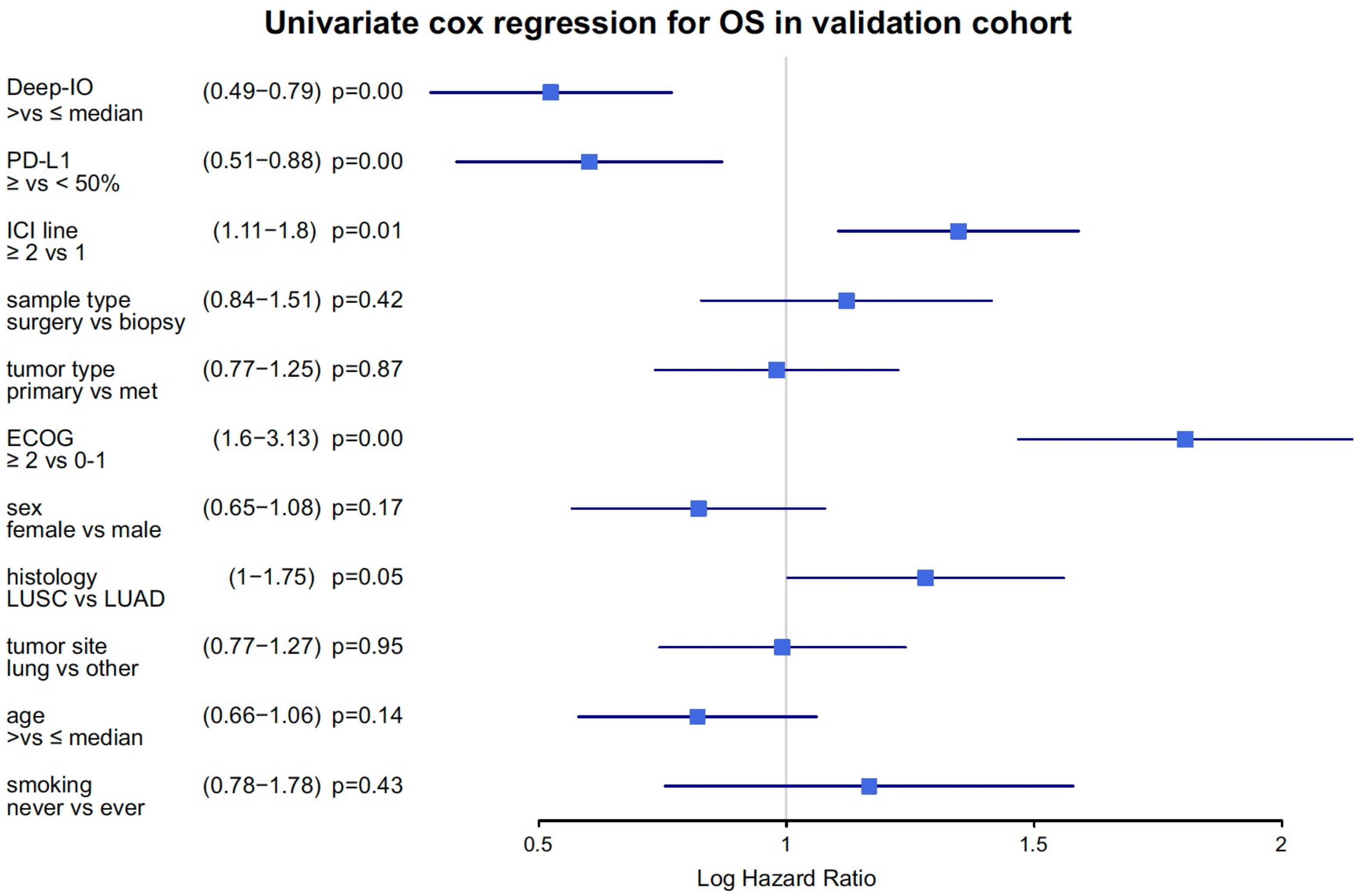

eFigure 17: Univariate analysis of OS: A forest plot displaying the results of a univariate analysis that identifies variables affecting overall survival (OS) to ICI treatment. Each row shows the hazard ratios (HR) and their corresponding 95% confidence intervals with horizontal lines. Number in parenthesis lower and upper 95% confidence intervals.

© 2024 Rakaee et al. JAMA Oncol.

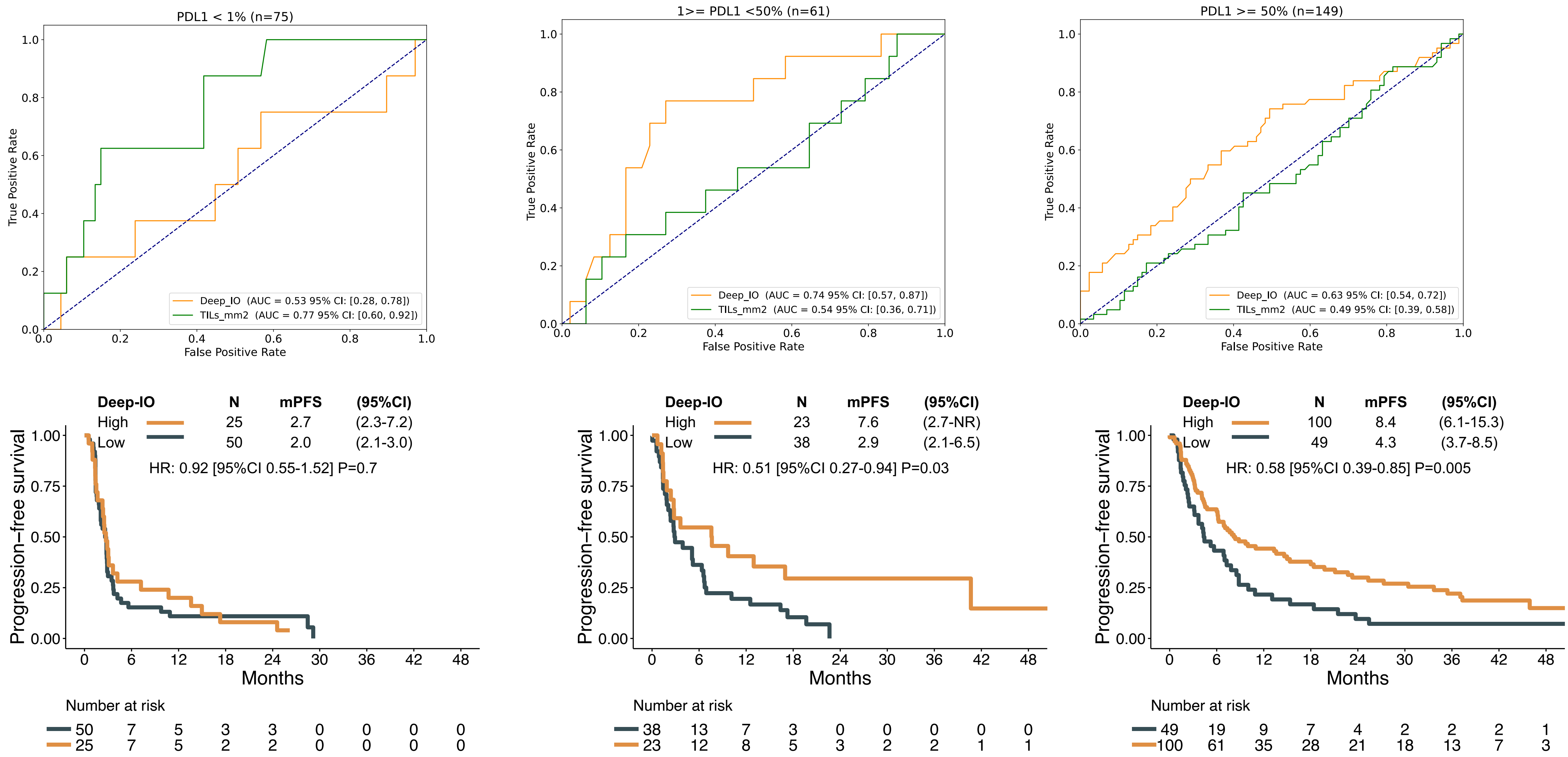

eFigure 18: Deep-IO vs TILs and ICI outcomes by PD-L1 subgroups. Comparative predictive power of Deep-IO versus TILs for the Objective Response Rate (ORR) to ICI across PD-L1 negative (<1%, left column), PD-L1 moderate (1-49%, middle column), and PD-L1 high (≥50%, right column) subgroups in the validation set. The lower row shows Progression-Free Survival (PFS) corresponding to Deep-IO scores in response to ICI therapy within these PD-L1 subgroups. HR, hazard ratio; CI, confidence interval; mPFS, median PFS in months; NR, not reached.

© 2024 Rakaee et al. JAMA Oncol.

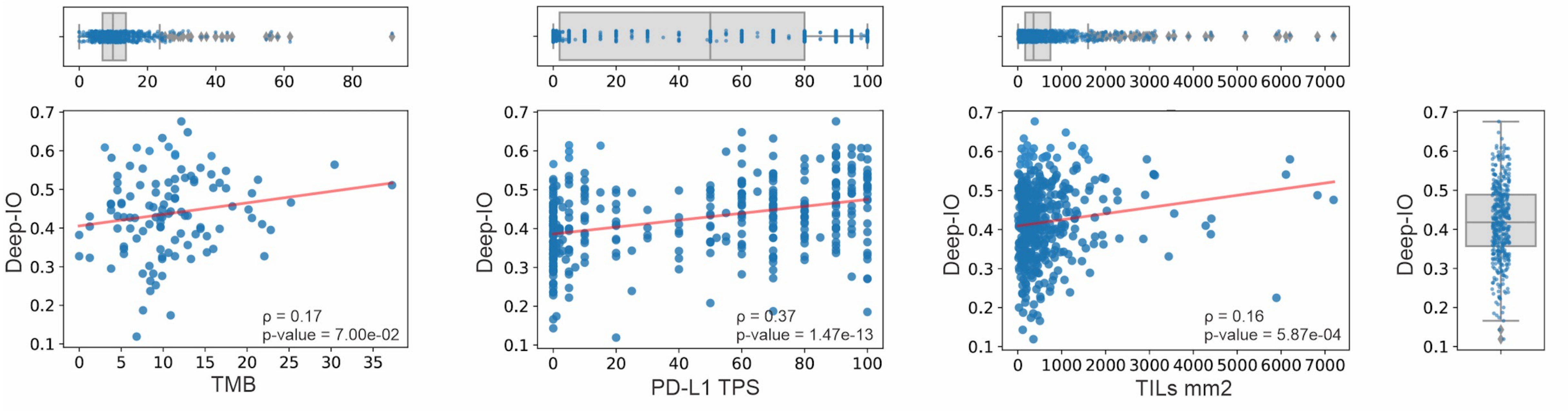

eFigure 19: Deep-IO vs other known biomarkers: Spearman's correlation scatter plots (with marginal box plots) comparing Deep-IO (n=437) with TMB (n=112), PD-L1 (n=364), and TILs (n=437) across the overall cohort including test (n=93) and validation (n=344) cohorts.

© 2024 Rakaee et al. JAMA Oncol.

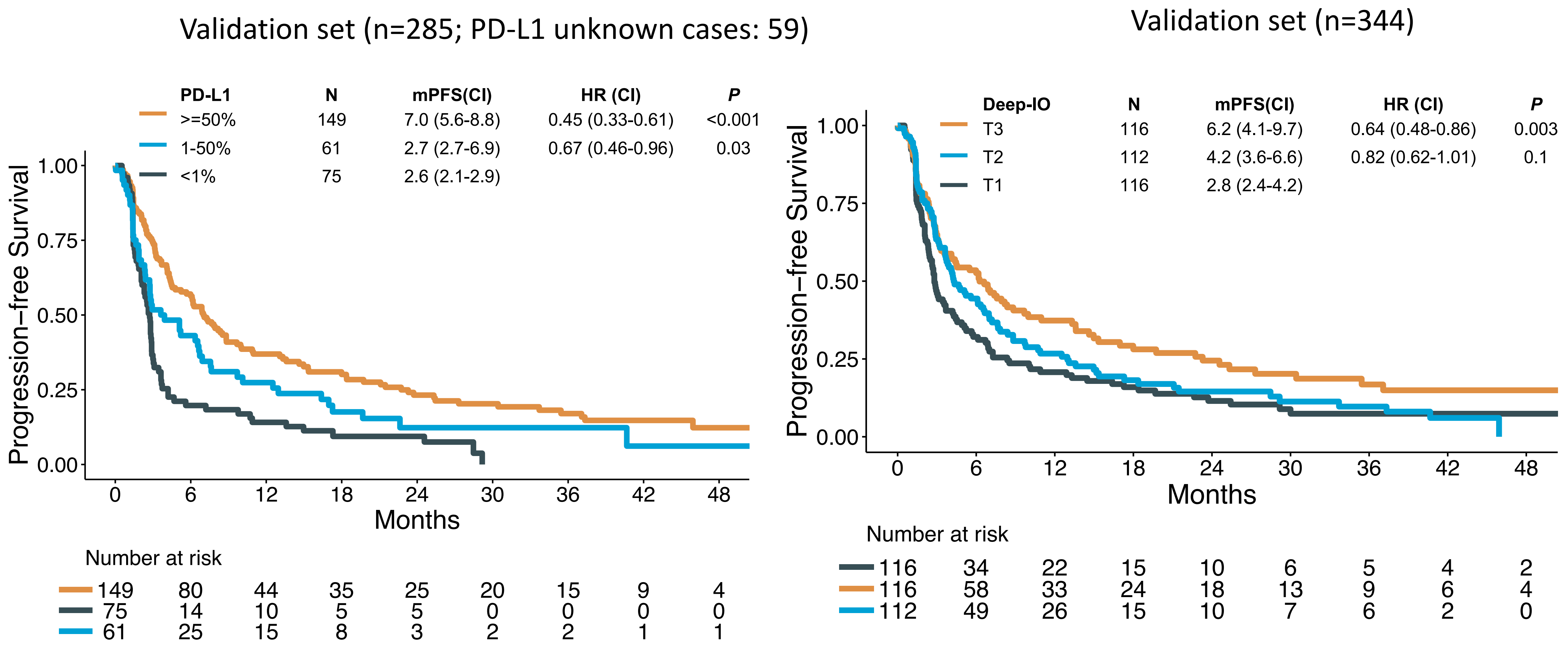

eFigure 20: Deep-IO vs PD-L1 and ICI outcome. Progression-Free Survival (PFS) stratified by PD-L1 using the standard cutoff (left KM curve) and by Deep-IO using tertile cutoffs (right KM curve) in the validation set. PD-L1 data were missing for 59 patients.Abbreviations: mPFS, median PFS in months; HR, hazard ratio; CI, confidence interval; T1, lower tertile; T2, middle tertile; T3, upper tertile.

© 2024 Rakaee et al. JAMA Oncol.

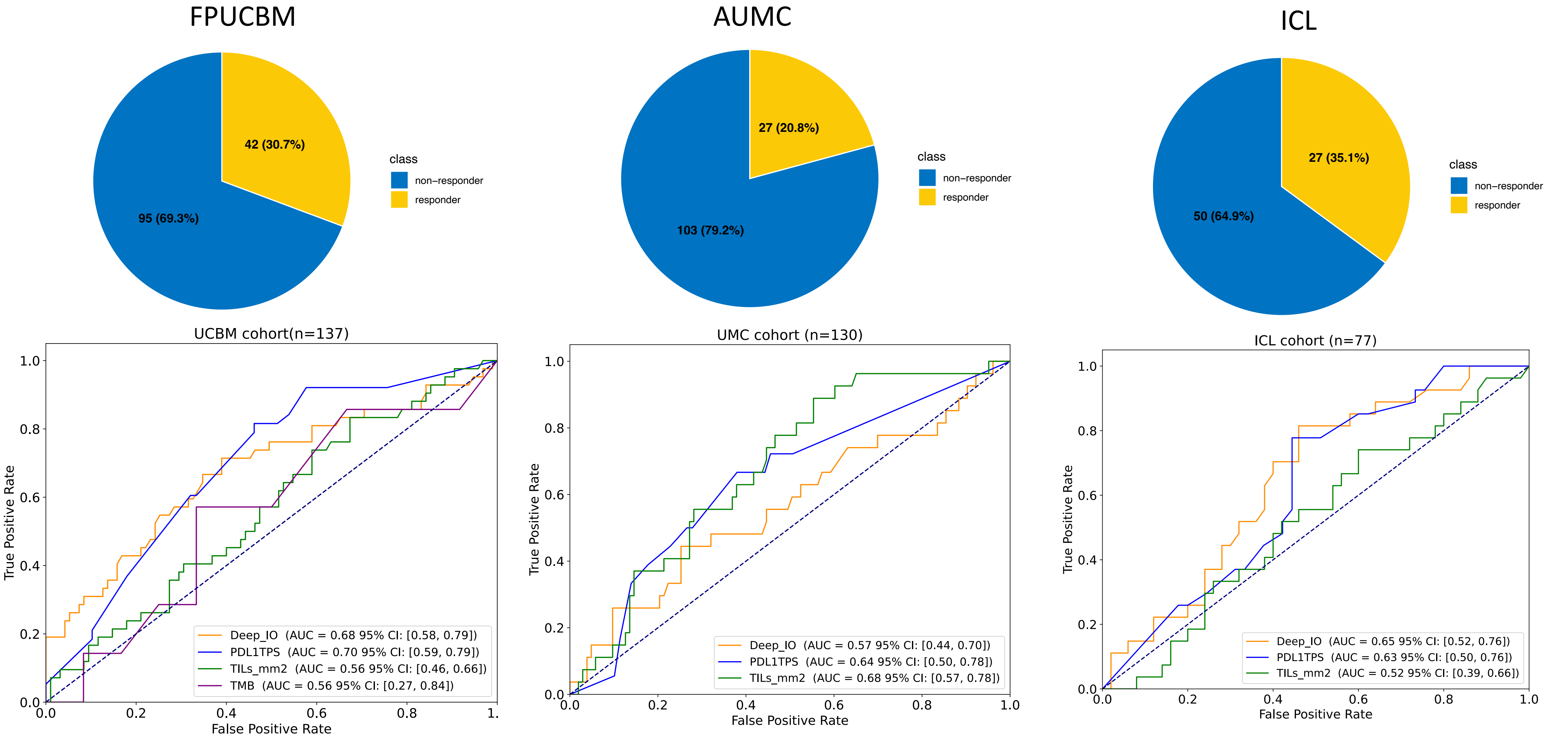

eFigure 21: ORR and performance power in validation subsets. The upper row pie charts represent the response rates in validation subsets. The lower ROC curves indicate the power of individual predictive biomarkers in differentiating responders from non-responders. In the FPUCBM sub-cohort, TMB was available for only 19 patients. CI, confidence interval.

© 2024 Rakaee et al. JAMA Oncol.

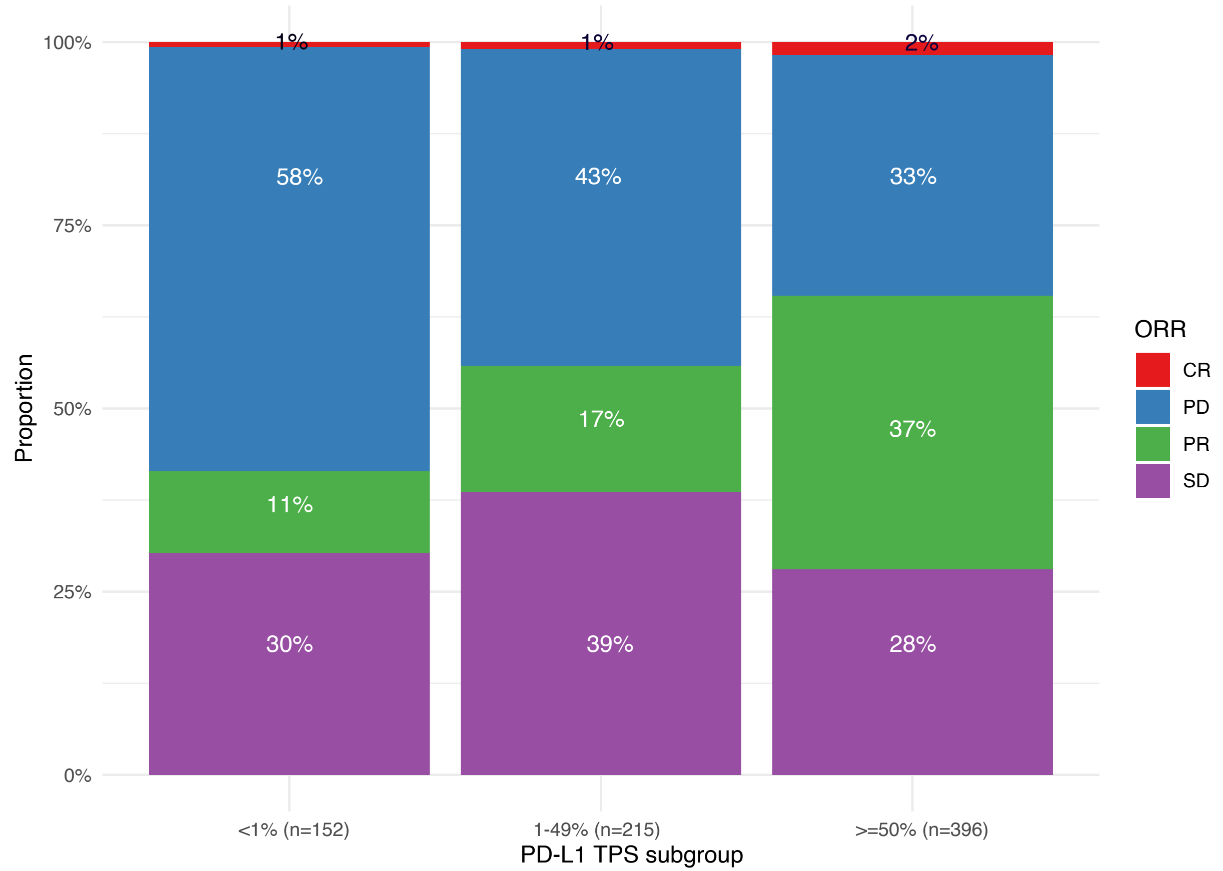

eFigure 22: **Response rate in overall material:** Objective response rate to ICI across PD-L1 subgroups in overall developmental and validation sets.

© 2024 Rakaee et al. JAMA Oncol.
