# Supplementary material for: Deep Learning Model for Predicting Immunotherapy Response in Advanced Non−Small Cell Lung Cancer
Source: JAMA Oncol. 2024 Dec 26;11(2):109–18. doi: 10.1001/jamaoncol.2024.5356 (PMC11843371; doi:10.1001/jamaoncol.2024.5356)
Supplement: Supplement 2. — Data Sharing Statement [file jamaoncol-e245356-s002.pdf]

Data Sharing Statement

Rakaee. Deep Learning Model for Predicting Immunotherapy Response in Advanced Non–Small Cell Lung Cancer. *JAMA Oncol*. Published December 26, 2024. doi:10.1001/jamaoncol.2024.5356

Data
Data available: No

Additional Information
Explanation for why data not available: Requests for sharing the clinical data should be directed to the corresponding author by non-commercial entities and must be reasonable. Due to the multi-centre nature of the study, data sharing will require approval from the principal investigator at each contributing center
